# Piezo1 specific deletion in endothelial cell protects the progression of pulmonary fibrosis in mice

**DOI:** 10.1186/s12964-026-02758-7

**Published:** 2026-02-28

**Authors:** Bin He, Qiaorui Tan, Honglin Xu, Xiaoting Chen, Rentao Wan, Youfen Yao, Xianmei Pan, Silin Liu, Xin Chen, Jintao Jiang, Shangfei Luo, Yajuan An, Jing Li

**Affiliations:** 1 Lingnan Medical Research Center, Guangzhou University of Chinese Medicine, Guangzhou, 510405 China; 2 The First Affiliated Hospital, Guangzhou University of Chinese Medicine, Guangzhou, 510405 China; 3 Innovation Research Center, Shandong University of Traditional Chinese Medicine, Jinan, 250307 China

**Keywords:** Piezo1, Pulmonary fibrosis, EndMT, Ca^2+^, Calpain

## Abstract

**Background:**

Piezo1, a mechanosensitive cation channel, plays a pivotal role in the pathogenesis of fibrosis by promoting intracellular calcium ion (Ca^2+^) influx and activating the calcium-dependent cysteine protease calpain. Pulmonary fibrosis (PF) is a progressive and often incurable disease, with current treatment strategies primarily relying on antifibrotic agents to slow disease progression. Accumulating evidence suggests that interdiction of Piezo1-induced Ca^2+^ signaling may suppress epithelial–mesenchymal transition (EMT) and modulate myofibroblast activation, thereby ameliorating PF. However, whether endothelial Piezo1 contributes to PF and the underlying mechanisms remain elusive.

**Objectives:**

This study aims to investigate the role of endothelial Piezo1 in mediating the development of PF.

**Methods:**

A murine model of PF was established using bleomycin (BLM). Mice were sacrificed at 14 and 21 days post-administration. To investigate the role of Piezo1 in mediating endothelial–mesenchymal transition (EndMT) during PF, endothelial cell-specific Piezo1 knockout mice were generated. In parallel, in vitro experiments were conducted in which mesenchymal transition was induced by TGF-β1, followed by treatment with the Piezo1 activator Yoda1, the inhibitor GsMTx4 (a spider-venom peptide that blocks cationic mechanosensitive channels), and Piezo1-targeting siRNA to further validate the functional role of Piezo1.

**Results:**

In this study, we found that endothelial-specific deletion of *Piezo1* (*Piezo1*^*ΔCDH5*^) in a BLM-induced PF mouse model effectively alleviates lung injury by reducing fibrotic lesions. In both in vivo and in vitro experiments, endothelial *Piezo1* knockout significantly inhibited EndMT. Phenotypically, *Piezo1*^*ΔCDH5*^ mice exhibited markedly reduced PF and inflammation. Mechanistically, endothelial Piezo1 senses mechanical cues within the pulmonary microenvironment and opens to elicit Ca^2+^ influx. The resultant rise in intracellular Ca^2+^ activates calpain, which subsequently amplifies p38 and ERK phosphorylation, thereby driving EndMT and contributing to PF.

**Conclusions:**

Our findings demonstrate that Piezo1 regulates the p38/ERK-MAPK signaling pathway via the Ca^2+^/calpain axis to inhibit EndMT, thereby ameliorating PF. These results suggest that Piezo1 may serve as a potential therapeutic target for slowing the progression of PF.

**Supplementary Information:**

The online version contains supplementary material available at 10.1186/s12964-026-02758-7.

## Introduction

PF is a progressive and life-threatening lung disorder. It represents the end-stage manifestation of various interstitial lung diseases (ILDs) [1–3]. It is characterized by irreversible lung tissue remodeling due to excessive extracellular matrix (ECM) deposition and persistent inflammation [4]. Idiopathic pulmonary fibrosis (IPF) is strongly connected to numerous risk factors, such as chronic inflammation, repeated infections, and exposure to environmental agents. These factors contribute to progressive ILD, leading to a gradual decline in pulmonary function and, ultimately, respiratory failure or death. The median survival following diagnosis is less than five years [5–9]. Although currently available anti-fibrotic therapies can slow disease progression, none achieve complete remission [10, 11]. Therefore, elucidating the underlying pathogenesis of PF and developing novel therapeutic strategies remain urgent clinical priorities.

Recent studies indicate that pulmonary endothelial cells might act as a potential origin of lung fibroblasts via the process of EndMT, which plays in the progression of fibrosis and represents a distinct biological process characterized by the downregulation of endothelial markers, and upregulation of mesenchymal markers [12]. This process is further defined by morphological transformation and the acquisition of a mesenchymal phenotype [13, 14]. This process has been implicated in fibrotic pathogenesis across multiple organs, including the heart, kidneys, and skin [15–17]. Under pathological conditions such as hypoxia, hypertonic stress, or mechanical injury, damaged endothelial cells may detach from the vascular endothelium, migrate into the interstitial space, and differentiate into activated fibroblasts. These fibroblasts, in turn, contribute to excessive ECM deposition, leading to fibrotic scarring and disruption of pulmonary architecture [18, 19]. Additionally, EndMT-derived cells exhibit increased migratory and invasive capabilities, while pulmonary microvascular endothelial injury enhances vascular permeability, facilitating pro-inflammatory factor infiltration into the interstitium and alveolar spaces [20, 21]. This cascade further exacerbates fibrogenesis through multiple pathways [22, 23]. Although EndMT inhibition has demonstrated therapeutic potential in ameliorating lung injury and fibrosis, its precise molecular mechanisms remain incompletely understood [24–26].

Piezo1, a mechanosensitive cation channel, has recently gained attention for its involvement in fibrotic diseases, including pulmonary, renal, and hepatic fibrosis [27–30]. Existing studies demonstrate that Piezo1 attenuates vascular remodeling in pulmonary arterial hypertension through EndMT inhibition, yet its role in PF remains undefined [31]. Given these findings, this study aims to determine whether Piezo1 deficiency alleviates PF by suppressing EndMT and fibroblast proliferation. In both the BLM-induced mouse model of PF and the in vitro EndMT model established by stimulating HUVECs with TGF-β1 [3], our rigorous experimental analyses revealed a significant upregulation of Piezo1. Concurrently, the expression levels of endothelial markers were markedly downregulated, mesenchymal markers were robustly induced, endothelial barrier integrity was compromised, and inflammatory infiltration was exacerbated. However, upon genetic knockout of Piezo1 in endothelial cells or administration of a specific Piezo1 inhibitor, these aberrant changes were substantially reversed. Collectively, these findings indicate that endothelial Piezo1 exerts a critical regulatory role in the pathogenesis of PF.

## Materials and methods

### Conditional Piezo1 knockout mice

All experimental animals were housed in a specific pathogen-free (SPF) environment with lighting conditions regulated to a 12-h light/dark rhythm. The Cre-loxP recombination system was utilized to generate mice with conditionally inactivated genes. Transgenic mice expressing Cdh5 (PAC)-CreERT2 (No. 029213, C57BL/6J background) and *Piezo1*^*fl/fl*^* (*from Jackson Laboratories, C57BL/6J background), generously supplied by Ralf Adams from the University of Munster in Germany. To activate Cre recombinase and induce gene deletion, 6–8-week-old mice were administered tamoxifen (Sigma, T5648, 75 mg/kg) via intraperitoneal injection for five consecutive days. Experimental procedures were carried out 7 days after the last injection.

### Pulmonary fibrosis model in mice

Mice were randomly allocated into two experimental groups: the BLM-induced PF group and the sham-operated control group. Following anesthesia induction, the cervical region was prepared by hair removal and disinfection. A midline incision was then carefully made along the neck using ophthalmic scissors. Blunt dissection was performed to expose the trachea. Throughout the experimental protocol, animals in the treatment cohort were given an intratracheal administration of BLM at a dose of 2 mg per kilogram, delivered in a 50 μl volume. Following instillation, the syringe was removed, and the mice were gently rotated laterally to facilitate uniform distribution of the drug within the lungs. The general activity of the mice was monitored daily, and body weight changes as well as mortality rates were recorded. For rescue experiments, a stock solution of GsMTx4 (Med Chem Express) was prepared in ddH_2_O. Mice subsequently received a 10 mg/kg intraperitoneal injection of this solution at a concentration of 5 μM from 7 to 21 days after BLM administration.

### Euthanasia and bronchoalveolar lavage fluid collection

On days 14 and 21 of the experiment, mice from each group were euthanized. Anesthesia was induced through intraperitoneal administration of 1% pentobarbital sodium. The mice were then positioned supine and fixed on the operating table. A plastic catheter was then inserted into the trachea, and the right main bronchus was ligated. Through the catheter, 0.5 mL of PBS was slowly instilled into the lungs, followed by three repeated washes to collect bronchoalveolar lavage fluid (BALF). The supernatant was harvested for the assessment of cytokines and other biochemical indicators, while the cell pellets were subjected to analysis using a cell counter.

### H&E staining procedure

Paraffin-embedded tissue sections were sequentially treated with xylene and graded ethanol solutions to remove paraffin wax and achieve complete dehydration. After dehydration, the sections were washed under flowing water. Next, they were treated with hematoxylin for staining purposes. Differentiation was performed by immersing the sections in 1% acid alcohol for 10 s, followed by another 3-min rinse with running water. After differentiation, the tissue slices were subjected to counterstaining with a 0.5% eosin solution for 2 min. Post-staining, the sections underwent dehydration through a series of progressively concentrated ethanol baths. They were then made transparent by soaking in xylene. Ultimately, the prepared sections were coverslipped and examined using a bright-field upright fluorescence microscope to capture images.

### Masson

The paraffin-embedded tissue sections were sequentially immersed in xylene solutions I and II for 10 min each to complete the dewaxing process. Subsequently, the sections were transferred sequentially into gradient ethanol, with each dehydration step lasting 5 min. Following dehydration, the slides were sequentially processed through: hematoxylin staining solution, running water rinse, 1% acid-alcohol differentiation, running water wash, eosin staining solution, and final running water rinse. Subsequently, the sections were sequentially immersed in 1% acetic acid solution and 1% phosphomolybdic acid solution, each for 3 min. Ultimately, the slides underwent dehydration, were cleared with xylene, and subsequently mounted using resin. Image acquisition was performed independently by an evaluator blinded to the experimental groups to ensure the objectivity of the data.

### Lung injury score

Two independent investigators evaluated each hematoxylin and eosin (H&E)-stained section separately. Using a microscope at 400 × magnification, they examined 300 alveoli per section and calculated the lung injury score based on a predefined scoring system that encompassed multiple parameters [32].

### Ashcroft scale

According to the Ashcroft method, we performed a semi-quantitative assessment of the degree of PF on Masson-stained lung sections. This scoring system categorizes the fibrotic status of lung tissue into grades ranging from 0 to 8, with each grade corresponding to a score from 0 to 8, where 0 represents normal lung architecture and 8 indicates complete fibrotic obliteration.

### ELISA

After thawing on ice, the BALF was centrifuged at 4 °C (2000 × g for 10 min), and the supernatant was collected and stored at low temperature. Measurements were performed using an ELISA kit (ThermoFisher, USA), adhering meticulously to the manufacturer’s instructions.

### Immunofluorescence

Following dewaxing in xylene (10 min) and graded ethanol dehydration. To improve cellular membrane permeability, tissue sections were treated with 0.2% Triton X-100 at ambient temperature. After permeabilization, a blocking step using 10% goat serum was carried out for one hour to prevent nonspecific antibody interactions. The sections were probed with Anti-Piezo1 antibody (1:500, Proteintech, China), Anti-CollagenI antibody (1:200, CST, USA), Anti-CollagenI antibody (1:200, CST, USA), Anti-Vimentin antibody (1:200, Abcam, UK), Anti-Fibronectin antibody (1:200, Abcam, UK), Anti-α-SMA antibody (1:200, Abcam, UK), Anti-VE Cadherin antibody (1:200, Abcam, UK), Anti-CD31 antibody (1:200, Abcam, UK), Anti-TGF beta 1 antibody (1:500, Proteintech, China), Anti-F4/80 antibody (1:200, Servicebio, China) primary antibody overnight at 4 °C (details provided in Supplementary Table S2). After the application of primary antibodies, the tissue slides were subsequently incubated with appropriate secondary antibodies conjugated to fluorescent dyes (Supplementary Table S2) at room temperature (RT). Nuclear staining was performed by incubating the sections with DAPI (Sigma Aldrich, USA) for 5 min. Slides were subsequently mounted with Fluoromount-G mounting medium (SouthernBiotech, USA) and sealed with coverslips. Imaging was performed using a Leica M165 FC confocal microscope system equipped with appropriate filters. Quantification was performed using ImageJ software.

### Wound healing assay

HUVECs proliferated until reaching 70%–80% cell confluence, at which point they were treated with the corresponding drug interventions. We performed the wound healing assay when cell confluence typically reached 90%–100% after 48 h of drug treatment. Briefly, a uniform scratch was created by a 200-μL pipette tip at the bottom of each dish. After rinsing the wells three times with PBS to eliminate dislodged cells. Photographs were taken at intervals of 0, 12, and 24 h using a Cytation5 microscope (Biotek, USA). The width of the wound was determined using ImageJ software, by measuring the distance between two parallel lines, which helped evaluate the cells’ capacity to migrate.

### Single-cell suspension preparation and flow cytometry analysis

Mice were euthanized and perfused on day 21 after the operation. The lung tissues were then excised, finely minced, and collected into Hank’s Balanced Salt Solution containing 1 mg/mL collagenase I (Gibco, USA). Tissue digestion was implemented at 37 °C with agitation at 180 rpm for 30 min. To isolate individual cells and eliminate any remaining tissue fragments, the digested solution was passed through a series of filters, first a 100-μm mesh and then a 40-μm mesh, both from Falcon in the USA. The filtered cell suspension was incubated on ice for 5 min with Red Blood Cell Lysis Buffer (Biosharp, China) to eliminate erythrocytes. After lysis, the cells were washed with HBSS, pelleted by centrifugation, and resuspended in the same buffer. The final cell density was adjusted to 1 × 10^6^ cells per 100 μL. To minimize nonspecific antibody binding, Fc receptors were subsequently blocked using Fc Block (BD Biosciences, USA) for 5 min prior to staining with the designated antibodies (detailed in Table S2). For stimulated cell samples, digestion with HBSS and two washes were performed prior to antibody staining (antibody information available in Table S2). All samples were analyzed by BD LSRF ortessa X-20 flow cytometer.

### Isolation and culture of primary murine pulmonary endothelial cells

After deep anesthesia with isoflurane, mice were perfused via the right ventricle with ice-cold sterile DPBS until the pulmonary circulation was completely cleared of blood, and the lungs were then excised en bloc. Trachea, main bronchi, and macroscopically visible large vessels were removed, and the remaining parenchyma was minced into ~ 1 mm^3^ fragments. The fragments were incubated for 30 min at 37 °C under 120 rpm agitation in digestion buffer containing 1 mg mL^-1^ collagenase I/II (1:1 ratio). The digested material was filtered through a 70 μm cell strainer, and the filtrate was centrifuged at 400 × g for 10 min at 4 °C; the pellet was resuspended to yield a single-cell suspension. Endothelial cells were purified by positive selection using a CD144 (VE-cadherin) immunomagnetic bead system. Briefly, the single-cell suspension was incubated with anti-mouse CD144 microbeads for 20 min at 4 °C in the dark, passed through a pre-cooled LS column, and subjected to a magnetic field. CD144^-^ cells were eluted; after removal of the magnet, CD144^+^ cells were flushed out with ice-cold buffer (purity > 95%). Purified cells were seeded at 5 × 10^5^ cells cm^-2^ in six-well plates pre-coated with 0.1% gelatin and cultured in complete endothelial cell medium at 37 °C, 5% CO_2_, and 95% humidity.

### Cell culture

HUVECs were kept in a moisture-controlled environment at 37 °C with a 5% CO_2_ atmosphere. Cells between the second and seventh passages were selected for further experimental procedures. When cells confluence reached approximately 70%, they were transferred to a low-serum medium containing 0.25% FBS and incubated in a basal medium with TGF-β1 (10 ng/mL; PeproTech, Rocky Hill, New Jersey, USA). Cells were assigned into different experimental groups: TGF-β1 group, TGF-β1 + GsMTx4 (30 nM; MedChemExpress) group, and TGF-β1 (10 ng/mL) + Yoda1 (5 nM; MedChemExpress) group. Cells were harvested for further analysis after treatment for 48 h.

### Electrophysiological recording and mechanical stimulation

The recordings of whole-cell mechanosensitive currents were conducted with the standard patch-clamp method. Pipettes for recording were pulled in two stages from borosilicate glass capillaries and fire-polished; their resistance ranged from 3 to 5 MΩ when filled with an internal solution composed of the following (in mM): 66 CsOH, 66 L-glutamate, 40 EGTA, 10 HEPES, 17 CaCl_2_, 2 MgCl_2_, 8 NaCl, and 1 Na_2_ATP, with pH adjusted to 7.2 using CsOH. The external bathing solution was formulated as (in mM): 130 NaCl, 5 KCl, 1.5 CaCl_2_, 1.2 MgCl_2_, 8 D-glucose, and 10 HEPES, with pH adjusted to 7.4 using NaOH. Offline correction was applied for liquid-junction potentials. An Axon Multiclamp 700B amplifier (Molecular Devices) was used to amplify the signals, which were then low-pass filtered at 1 kHz and sampled at 3 kHz via a Digidata 1440 A digitizer. pCLAMP software (Molecular Devices) was employed for data acquisition and subsequent analysis. Mechanical stimulation was delivered via a fire-polished, blunt-tipped glass probe positioned ~ 3 µm above the cell membrane at an angle of ~ 80°. The probe was driven by a P-601 piezo actuator (Physik Instrumente) programmed to generate square-pulse indentations. Each pulse produced a 1 µm step displacement toward the membrane with a 2 ms rise time, held for ~ 200 ms, and delivered at 10 s intervals. The mechanical threshold was defined as the smallest indentation that evoked a discernible current (amplitude exceeding baseline noise). All recordings were analyzed offline with Clampfit (Molecular Devices).

### Transfection procedure

Transfection was performed using Lipofectamine 3000 and Opti-MEM medium. When HUVECs reached approximately 90% confluence, 20 nM of siRNA and/or 0.5 μg of plasmid DNA were added for transfection. The *Piezo1* siRNA sequence used was GCAGCAUGACAGACGACAU with a non-targeting siRNA as the negative control. Following a 6-h transfection period, the cells were washed and provided with fresh medium for continued incubation. Two days following the transfection procedure, the success rate of the transfection was assessed by observing the cells under a fluorescence microscope and comparing them to a group that had not been transfected. After this evaluation, the cells that had undergone transfection were harvested 48 h after the transfection for use in subsequent experimental work.

### Intracellular calcium ion measurement

HUVECs were plated in a 96-well cell culture dish at 90% confluence. After removing the culture medium, the cells were incubated at 37 °C for 1 h in a detection buffer containing 2 μM Fura-2 AM and 0.01% Pluronic Acid (Sigma, USA). The composition of the detection solution included 10 mM HEPES, 130 mM sodium chloride, 1.2 mM magnesium chloride, 5 mM potassium chloride, 1.5 mM calcium chloride, and 8 mM D-glucose. (pH 7.4, adjusted with NaOH). Intracellular Ca^2+^ signaling was dynamically monitored using a FlexStation 3 microplate reader (Molecular Devices, USA). A dual-wavelength excitation mode at 340 nm and 380 nm was employed, and the fluorescence intensity ratio (340/380) at 510 nm was used to quantitatively analyze changes in intracellular calcium ion concentration.

### Calpain activity assay

After perfusion with 0.9% NaCl, lung tissues were excised and homogenized in the provided extraction buffer, then were centrifuged at 10,000 × g for 1 min. Protein levels in the collected supernatant were quantified using the Pierce BCA Protein Assay Kit. Calpain enzymatic activity was assessed following the manufacturer’s protocol using the Calpain Activity Assay Kit (Abcam, ab65308, UK). To prepare for measurement, 85 µL of extraction buffer was mixed with the supernatant, and the mixture was placed into a 96-well plate. Following this, each well received 5 µL of a substrate specific to calpain and 10 µL of a buffer designed for the reaction. The plate was subsequently kept at 37 °C, shielded from light, for a period of one hour. Following the incubation period, fluorescence was detected using a microplate reader configured with excitation at 400 nm and emission at 505 nm. Results were calculated by subtracting the background absorbance from the blank control. For calpain activity assessment in HUVECs, cells were collected in the provided extraction buffer, then the same procedure was performed as described above.

### RT-qPCR

Total RNA was isolated using TRIzol reagent (Invitrogen, Carlsbad, CA, USA) according to the manufacturer’s protocol, followed by DNase I treatment. Reverse transcription was performed using the Evo M-MLV RT Premix for RT-qPCR Kit (Accurate Biology, China) with 1 μg of total RNA as the template. RT-qPCR was carried out using the SYBR Green Premix Pro Taq HS RT-qPCR Kit (Accurate Biology, China) following the manufacturer’s instructions. Relative gene expression was calculated using the 2 (− ΔΔCt) method.

### Western blotting

For protein separation, loaded 20 μg of protein lysate onto an 8% SDS-PAGE gel for electrophoresis. Upon completion of the electrophoresis process, the gel was carefully relocated onto a PVDF membrane sourced from Millipore, USA. To prevent nonspecific binding, the membrane was treated with a blocking solution. It was then left to interact with the primary antibody throughout the night. Anti-p38 MAPK antibody (1:1000), Anti-p-p38 MAPK antibody (1:1000), Anti-p44/42 MAPK (ERK 1/2) antibody (1:1000), Anti-P-p44/42 MAPK (ERK 1/2) antibody (1:1000), and anti-GAPDH (1:1000) were from CST. The next day, the membrane underwent a series of washes with PBST solution before being exposed to the secondary antibody at room temperature for one hour. Following this step, the membrane was treated with ECL Western Blotting Detection Reagent from Millipore, USA, to enable chemiluminescent visualization. The final images were captured and analyzed using the ChemiScope imaging system manufactured by CLiNX, China. Finally, protein expression levels were quantified in ImageJ software with the results normalized to the internal control (β-actin/GAPDH).

### Data analysis

Origin Pro 2025 was utilized for conducting statistical evaluations. Continuous variables are reported as averages accompanied by their standard deviations (Mean ± SD), where ‘n’ indicates the total count of separate experimental trials, equating to the repetitions for each individual well. Two-tailed Student’s t test were used in comparisons between two groups, while ANOVA analysis with followed by Dunnett test or Tukey’s test were compared using among over two groups. When datasets exhibited deviations from normal distribution or displayed heterogeneity of variance, non-parametric statistical methods were employed. Results were deemed statistically significant if the *p*-value fell below 0.05 (*P* < 0.05), and such findings were marked with an asterisk.

## Results

### Piezo1 is elevated in the vascular endothelium of pulmonary fibrosis lesions

**Fig. 1:**
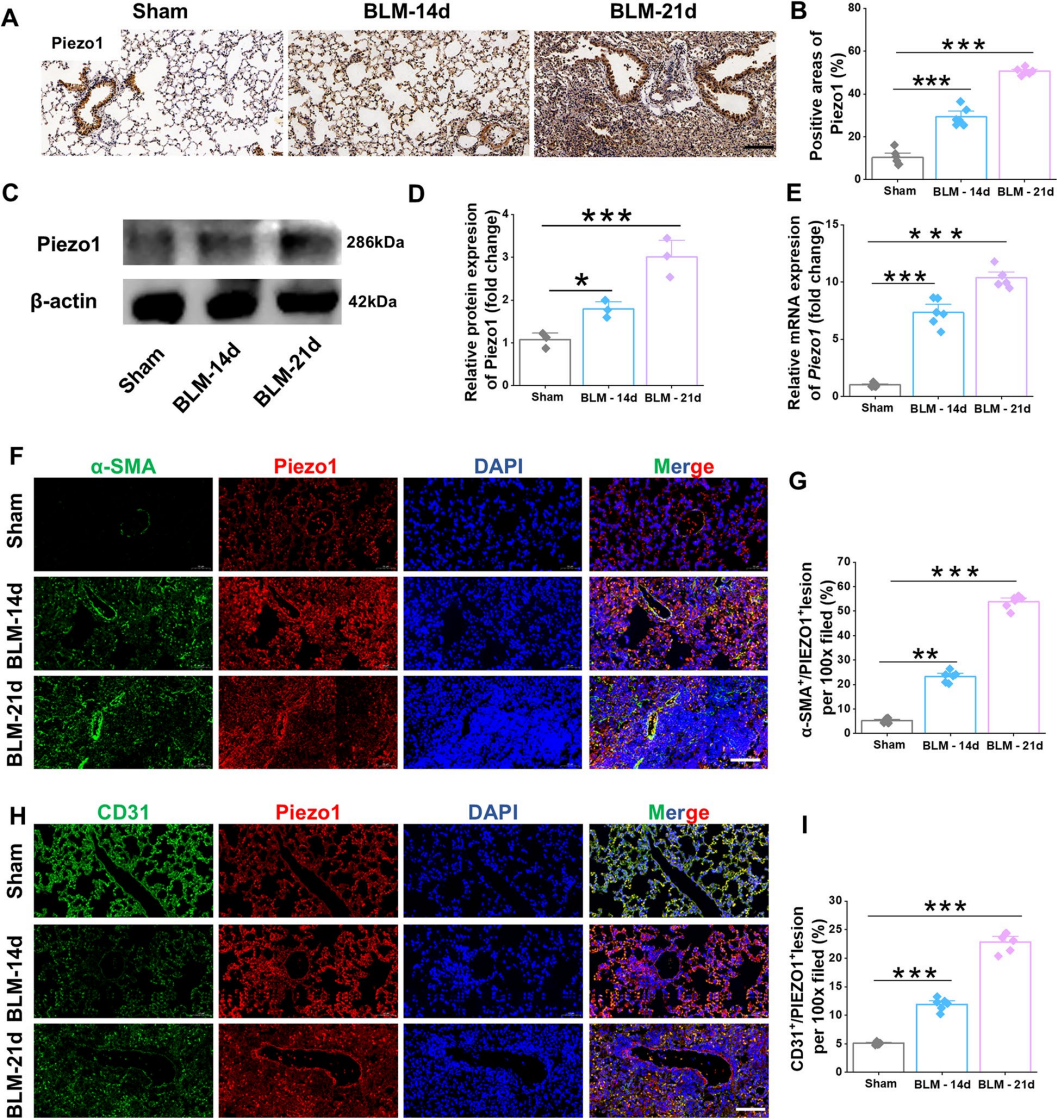
Piezo1 is upgraded in BLM-induced pulmonary fibrotic mice. **A** Images and **B** quantification of immunohistochemistry staining with Piezo1 in lung sections (*n* = 6). Scale bar, 50 μm. **C** Western blot analysis and **D** quantification of the protein expression of Piezo1 in PMECs (*n* = 3). **E** Relative mRNA expression of *Piezo1* in lung tissues (*n* = 6). **F** Dual immunofluorescent staining targeting on α-SMA (green) and Piezo1 (red), and **G** its quantification in lung sections (*n* = 6). Scale bar, 50 μm. **H** Dual immunofluorescent staining targeting on CD31 (green) and Piezo1 (red), and **I** its quantification in lung sections (*n* = 6). Scale bar, 50 μm. Lung tissues, paraffin sections, and PMECs were obtained from C57BL/6J mice subjected to BLM (14/21 days) or sham operation. Data are presented as mean ± SEM. *P < 0.05, **P < 0.01, ***P < 0.001

Previous studies have shown that Piezo1 plays an important role in the pathophysiology of a variety of fibrotic diseases [22, 27, 29, 33, 34]. Increased Piezo1 expression has been observed in PF [35]. Immunohistochemical analyses were performed to investigate the expression pattern of Piezo1 during the progression of PF. The results demonstrated that Piezo1 expression was markedly upregulated in BLM-induced fibrotic lungs, with significant increases observed at postoperative days 14 and 21 (Fig. 1A-B). Consistently, Western blotting and RT–qPCR analyses further confirmed that both Piezo1 mRNA and protein levels were significantly elevated in lung tissues after BLM treatment compared with the sham group (Fig. 1C-E). In addition, an increased proportion of α-SMA–positive and Piezo1-positive cells was observed within fibrotic lesions (Fig. 1F-G). Moreover, the co-localization signals of Piezo1 with the endothelial marker CD31 were significantly enhanced at 14 and 21 days following BLM administration (Fig. 1H–I). Collectively, these findings indicate that Piezo1 activation in the pulmonary vascular endothelium is progressively enhanced in a time-dependent and pathology-associated manner during the development of PF.

### Endothelial-specific Piezo1 knockout mice were generated for pulmonary fibrosis studies

To explore the functional role of endothelial Piezo1 in PF, we created *Piezo1-CDH5-Cre*^+^ *(Piezo1*^*ΔCDH5*^) and *Piezo1-CDH5-Cre*^*−*^* (Piezo1*^*fl/fl*^) mice by crossing *Piezo1*^*fl/fl*^ mice with a transgenic line that expresses tamoxifen-inducible, endothelial cell (EC)-specific Cre recombinase under the control of the CDH5 promoter. *Piezo1* knockout were confirmed by genotyping with gel electrophoresis, followed by RT-qPCR and Yoda1-induced calcium influx assays in liver endothelial cells isolated from *Piezo1*^*ΔCDH5*^ and *Piezo1*^*fl/fl*^ mice (Fig. S1A-E). Since systemic knockout of Piezo1 in endothelial cells leads to embryonic lethality, this study employed a tamoxifen-inducible CreERT2 system to achieve endothelial cell-specific conditional knockout of Piezo1 in adult mice.

**Fig. 2:**
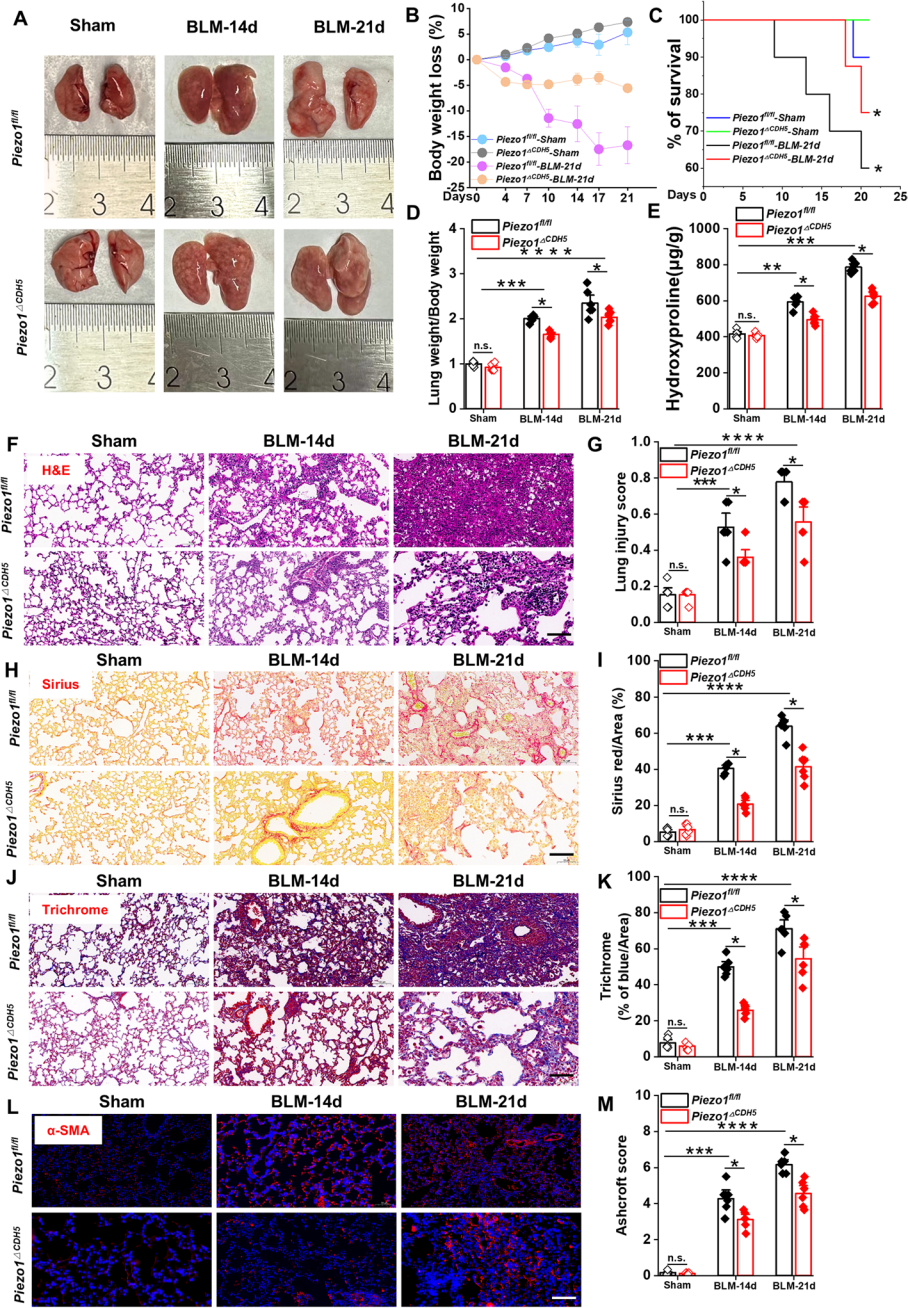
EC-specific Piezo1 deficiency reduces lung injury and pulmonary fibrosis. **A** Gross lung morphology of each group (*n* = 6). **B** Weight loss percentage (*n* = 6), **C** survival rate (*n* = 10), **D** pulmonary weight ratio (*n* = 6) and **E** level of hydroxyproline in lung tissue (*n* = 6) are measured in each group. **F** H&E staining images and **G** lung injury score of each group (*n* = 6). Scale bar, 50 μm. **H** Images and **I** quantification of collagen positive areas with Sirius red staining (*n* = 6). Scale bar, 50 μm. **J** Images and **K** quantification of collagen positive areas with Masson’s trichrome staining (*n* = 6). Scale bar, 50 μm. **L** Images of immunofluorescence staining with α-SMA and **M** Ashcroft score in lung sections (*n* = 6). Scale bar, 50 μm. Lung tissues and sections were obtained from *Piezo1*^*fl/fl*^ and *Piezo1*^*△CDH5*^ mice subjected to BLM (14/21 days) or sham operation. Data are presented as mean ± SEM. *P < 0.05, **P < 0.01, ***P < 0.001, ****P < 0.0001, ns, not significant

Photos of lung tissue revealed progressive alveolar edema in BLM-treated lungs over time (Fig. 2A). Survival analysis showed that *Piezo1*^*ΔCDH5*^ mice exhibited improved 21-day survival rate, reduced weight loss, and restored lung weight-to-body weight ratio compared with *Piezo1*^*fl/fl*^ mice (Fig. 2B-D). Additionally, hydroxyproline content in *Piezo1*^*ΔCDH5*^ lungs significantly decreased (Fig. 2E). Following histopathological evaluation revealed severe alveolar structural disorganization, inflammatory cell infiltration, and interstitial thickening in *Piezo1*^*fl/fl*^ mice at day 21. In contrast, *Piezo1*^*ΔCDH5*^ mice exhibited reduced alveolar septal thickness and inflammatory infiltration scores (Fig. 2F-G). Sirius red staining demonstrated decreased collagen deposition area (Fig. 2H-I). Masson’s trichrome staining and immunofluorescence staining targeting α-SMA confirmed lower collagen fiber density and fibrotic area in lungs from *Piezo1*^*ΔCDH5*^ mice (Fig. 2J-M). Collectively, these data demonstrate that endothelial Piezo1 deficiency attenuates PF through the preservation of alveolar integrity, suppression of aberrant collagen deposition, and modulation of inflammation.

### Endothelial-specific Piezo1 deletion attenuates endothelial barrier dysfunction and pulmonary inflammation

**Fig. 3:**
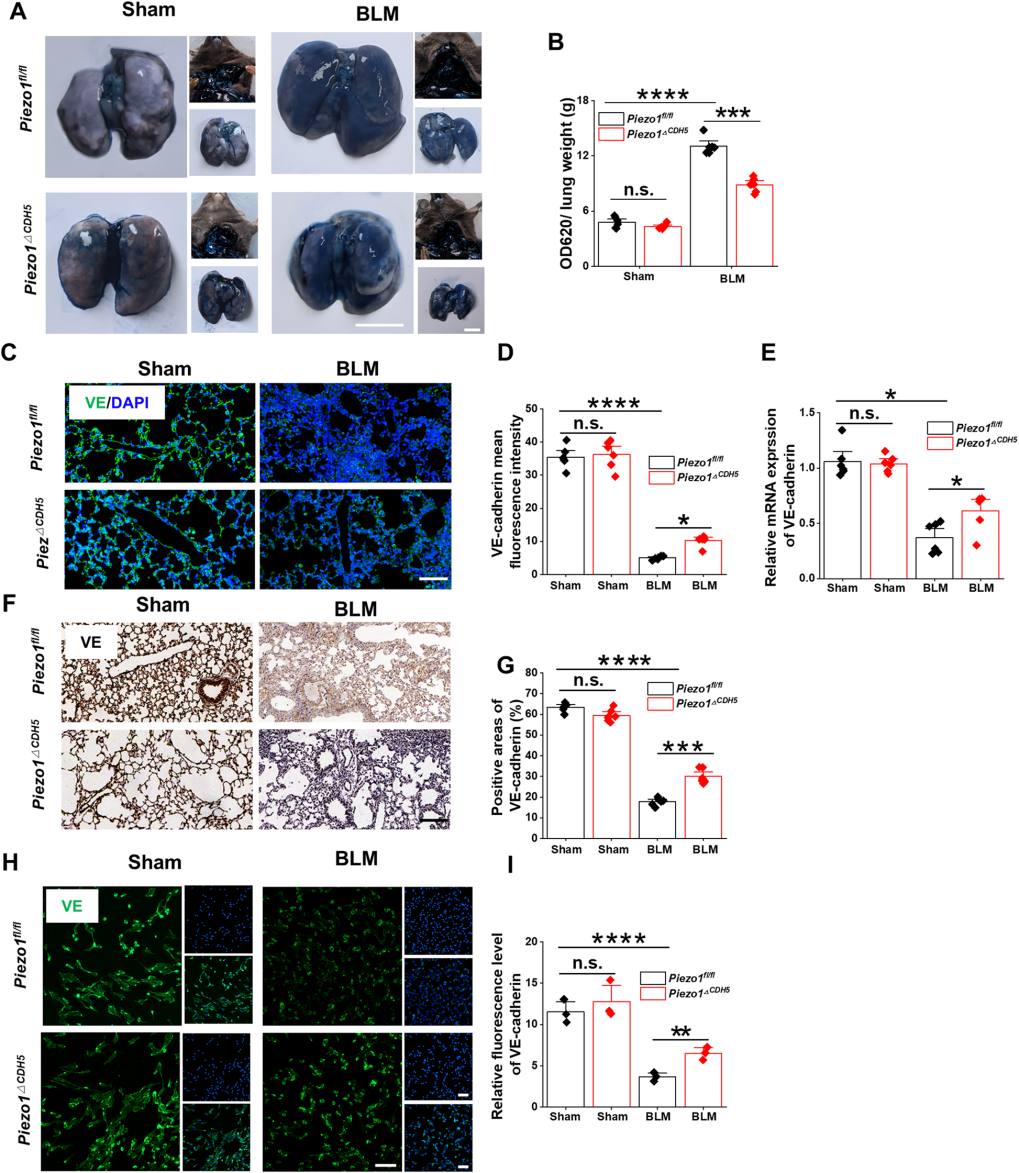
EC-specific Piezo1 deficiency preserves endothelial barrier integrity in pulmonary fibrosis. **A** Representative images and **B** quantification of evans blue-dye extravasation assay (*n* = 6). Scale bar, 1 cm. **C** Images and **D** quantification of immunofluorescent staining on VE-cadherin in lung sections (*n* = 6). Scale bar, 50 μm. **E** Relative mRNA expression of *Piezo1* in lung tissues (*n* = 6). **F** Images and **G** quantification of immunohistochemistry staining on VE-cadherin in lung sections (*n* = 6). Scale bar, 50 μm. **H** Images and **I** quantification of immunofluorescent staining on VE-cadherin in PMECs (*n* = 3). Scale bar, 50 μm. Lung tissues, paraffin sections, and PMECs were obtained from *Piezo1*^*fl/fl*^ and *Piezo1*^*△CDH5*^ mice subjected to BLM or sham operation. Data are presented as mean ± SEM. *P < 0.05, **P < 0.01, ***P < 0.001, ****P < 0.0001, ns, not significant

It is well established that the progression of PF is frequently accompanied by disruption of the endothelial barrier, which is characterized by the disassembly of adherens junctions such as VE-cadherin. This process facilitates transendothelial migration of inflammatory cells, thereby exacerbating pulmonary inflammation. Moreover, a sustained inflammatory microenvironment can further promote EndMT, ultimately accelerating fibrotic remodeling. To investigate the role of endothelial-specific Piezo1 deletion in alleviating BLM–induced PF, we systematically evaluated the inflammatory responses including histopathological alterations in lung tussues and inflammatory cytokine profiles in BALF. We first assessed endothelial permeability using the Evans Blue extravasation assay. BLM administration markedly increased pulmonary vascular permeability, whereas *Piezo1*^*ΔCDH5*^ mice exhibited significantly reduced Evans Blue leakage compared with *Piezo1*^*fl/fl*^ mice (Fig. 3A–B), indicating that endothelial Piezo1 deficiency preserves endothelial barrier integrity. Consistently, immunofluorescence staining, RT–qPCR, and immunohistochemical analyses of adherens junction–associated molecules revealed that the disruption of VE-cadherin–mediated junctions was substantially attenuated in the lungs of *Piezo1*^*ΔCDH5*^ mice relative to controls (Fig. 3C–G), providing molecular evidence for restored barrier function. To further validate these findings at the cellular level, primary pulmonary endothelial cells (PMECs) were isolated from fibrotic lungs exhibited a decreased barrier strength and subjected to immunofluorescence analysis. In PMECs derived from control mice, VE-cadherin displayed a continuous and uniform distribution along cell–cell borders. In contrast, endothelial cells from fibrotic lungs exhibited disorganized alignment, fragmented junctional structures, and irregular morphology. Notably, these pathological alterations were markedly ameliorated in endothelial cells isolated from *Piezo1*^*ΔCDH5*^ mice (Fig. 3H–I), further supporting a protective role of Piezo1 deficiency in maintaining endothelial structural integrity.

**Fig. 4:**
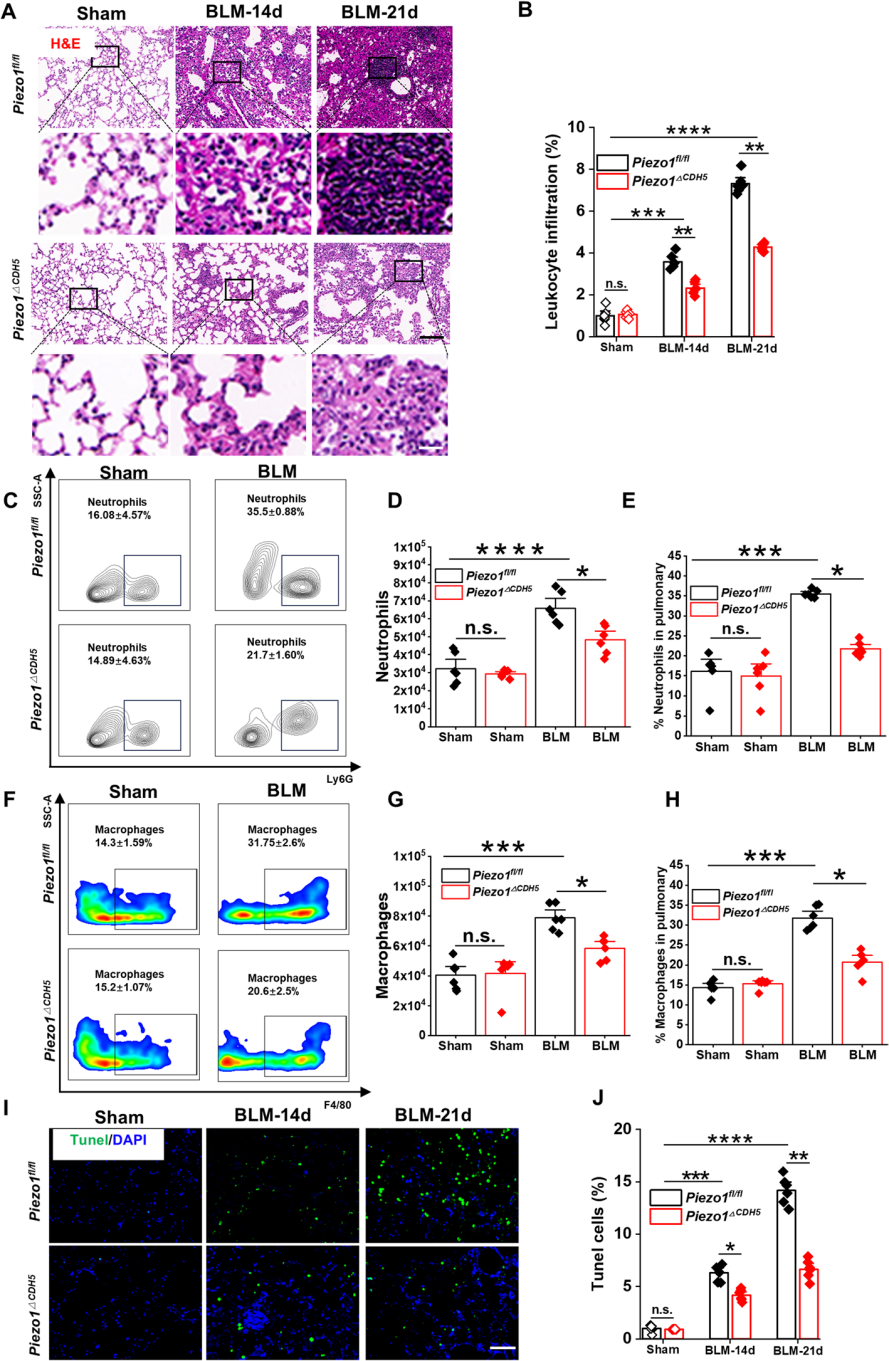
EC-specific Piezo1 deficiency reduces inflammatory cell infiltration in fibrotic lungs. **A** Representative H&E staining images and **B** the corresponding quantification analysis of leukocyte infiltration (*n* = 6). Scale bar, 50 μm. (enlarged images, Scale bar, 20 μm). **C** Flow cytometric plots of CD11b^+^Ly6G^+^, with statistical analysis on **D** the number and **E** the percentage of neutrophils from each group (*n* = 6).** F** Flow cytometric plots of CD11b^+^Ly6G^−^F4/80^+^, with statistical analysis on **G** the number and **H** the percentage of macrophages from each group (*n* = 6). **I** Representative TUNEL staining images and **J** quantificationin in lung tissue sections of each group (*n* = 6). Scale bar, 50 μm. Lung sections were obtained from *Piezo1*^*fl/fl*^ and *Piezo1*^*△CDH5*^ mice subjected to BLM (14/21 days) or sham operation. The lung single-cell suspension for flow cytometric analysis were prepared from *Piezo1*^*fl/fl*^ and *Piezo1*^*△CDH5*^ mice subjected to BLM for 21 days or sham operation. Data are presented as mean ± SEM. *P < 0.05, **P < 0.01, ***P < 0.001, ****P < 0.0001, ns, not significant

We next performed a multi-level analysis of inflammatory cell infiltration. H&E staining revealed a significant reduction in polymorphonuclear leukocyte accumulation in the lungs of *Piezo1*^*ΔCDH5*^ mice compared with control mice following BLM challenge (Fig. 4A–B). Flow cytometric analysis further quantified alterations in immune cell populations, demonstrating that BLM treatment significantly increased both the absolute numbers and proportions of neutrophils (CD11b^+^ Ly6G^+^) and macrophages (CD11b^+^ Ly6G-F4/80^+^) in the lung. Importantly, these increases were markedly attenuated in *Piezo1*^*ΔCDH5*^ mice (Fig. 4C–H). Consistent with these findings, immunofluorescence and immunohistochemical analyses showed significantly reduced infiltration of F4/80^+^ macrophages within fibrotic regions of *Piezo1*^*ΔCDH5*^ lungs (Fig. S2A–D).

At the level of inflammatory mediators, RT–qPCR analysis demonstrated that the mRNA expression of the pro-inflammatory cytokines was significantly downregulated in lung tissues from *Piezo1*^*ΔCDH5*^ mice (Fig. S2E–G). ELISA of BALF further revealed that protein levels of fibrosis-associated cytokines, including TNF-α, IL-1β and IL-6, were significantly reduced in *Piezo1*^*ΔCDH5*^ mice compared with controls (Fig. S2H–J). In parallel, BALF cellular analysis indicated that the BLM-induced increases in total cell counts, as well as the proportions of neutrophils and macrophages, were partially reversed in *Piezo1*^*ΔCDH5*^ mice (Fig. S2K–L). Furthermore, TUNEL assays revealed a significant reduction in apoptotic cells in the lung tissues of *Piezo1*^*ΔCDH5*^ mice (Fig. 4I–J), suggesting that endothelial Piezo1 deficiency may also contribute to the attenuation of PF by suppressing apoptosis.

Taken together, these results demonstrate that endothelial-specific deletion of Piezo1 markedly mitigates BLM-induced endothelial barrier disruption, suppresses inflammatory cell infiltration, reduces pro-inflammatory and pro-fibrotic cytokine production, and decreases pulmonary cell apoptosis. Collectively, these coordinated effects confer a multifaceted protective role against the development of PF.

### Endothelial *Piezo1* knockout alleviates pulmonary fibrosis

**Fig. 5:**
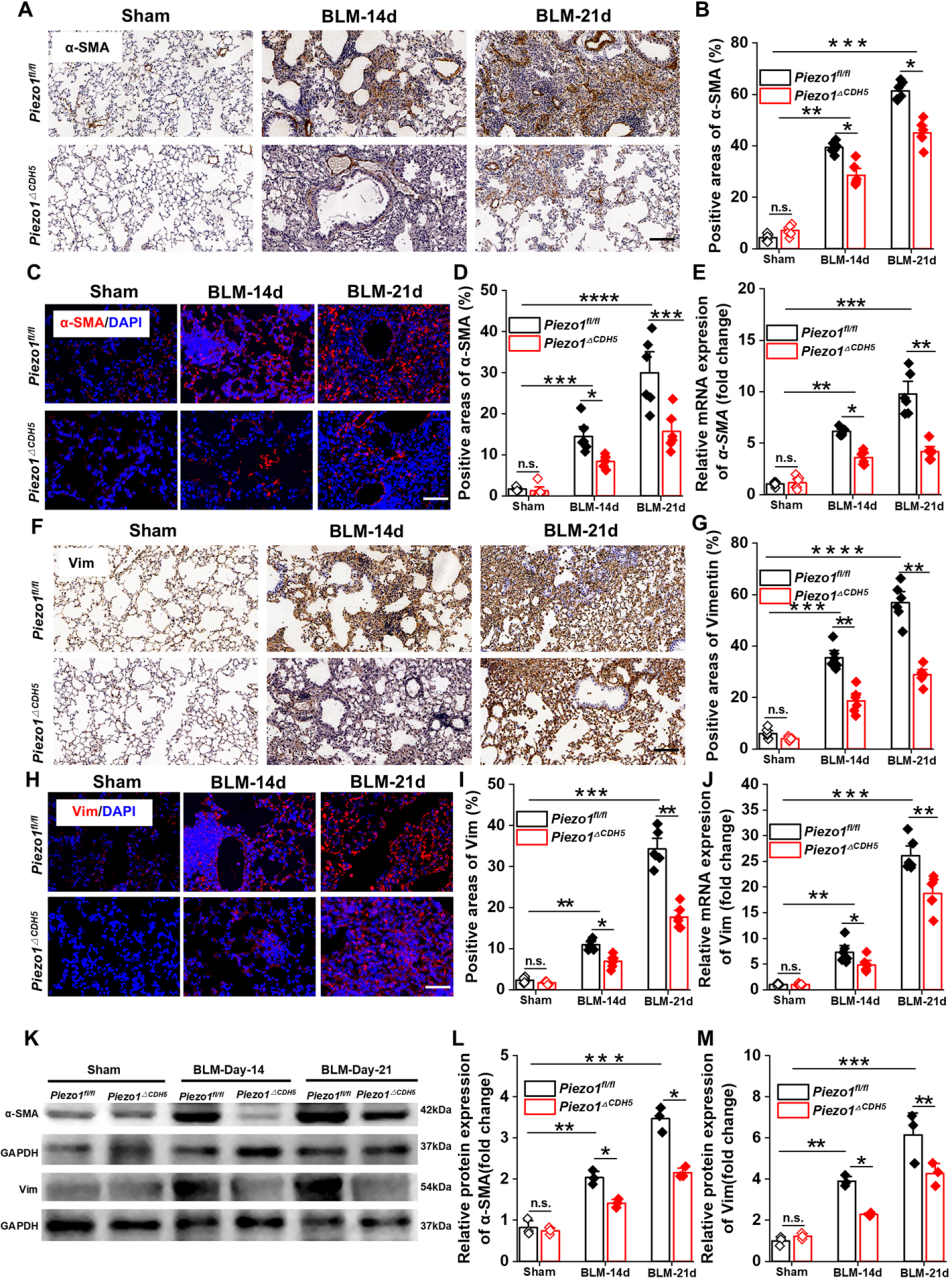
EC-specific *Piezo1* knockout alleviates pulmonary fibrosis. **A** Images and **B** quantification of immunohistochemistry staining with α-SMA in lung sections (*n* = 6). Scale bar, 50 μm. **C** Images and **D** quantification of immunofluorescent staining with α-SMA in lung sections (*n* = 6). Scale bar, 50 μm. **E** Relative mRNA expression of *α-SMA* in lung tissues (*n* = 6). **F** Images and **G** quantification of immunohistochemistry staining with Vim in lung sections (*n* = 6). Scale bar, 50 μm. **H** Images and **I** quantification of immunofluorescent staining with Vim in lung sections (*n* = 6). Scale bar, 50 μm. **J** Relative mRNA expression of *Vim* in lung tissues (*n* = 6). **K** Western blot analysis and **L-M** quantification of the protein expression of α-SMA and Vim in lung tissues (*n* = 3). Lung tissues and sections were obtained from *Piezo1*^*fl/fl*^ and *Piezo1*^*△CDH5*^ mice subjected to BLM (14/21 days) or sham operation. Data are presented as mean ± SEM. *P < 0.05, **P < 0.01, ***P < 0.001, ****P < 0.0001, ns, not significant

To elucidate the protective effects of endothelial Piezo1 deletion on PF, we systematically evaluated the impact of endothelial-specific Piezo1 deficiency on BLM–induced PF at both the protein and gene expression levels. Immunofluorescence staining, immunohistochemistry, RT–qPCR, and Western blot analyses were employed to assess the expression of fibrosis-associated molecular markers. Immunofluorescence and immunohistochemical analyses revealed that the protein expression levels of α-SMA and vimentin (Vim) were significantly reduced in the lung tissues of *Piezo1*^*ΔCDH5*^ mice compared with *Piezo1*^*fl/fl*^ control mice. Consistently, RT–qPCR analyses demonstrated a corresponding downregulation of *α-SMA* and *vimentin* mRNA expression (Fig. 5A–J). These findings were further corroborated at the protein level by Western blot analysis (Fig. 5K–M).

**Fig. 6:**
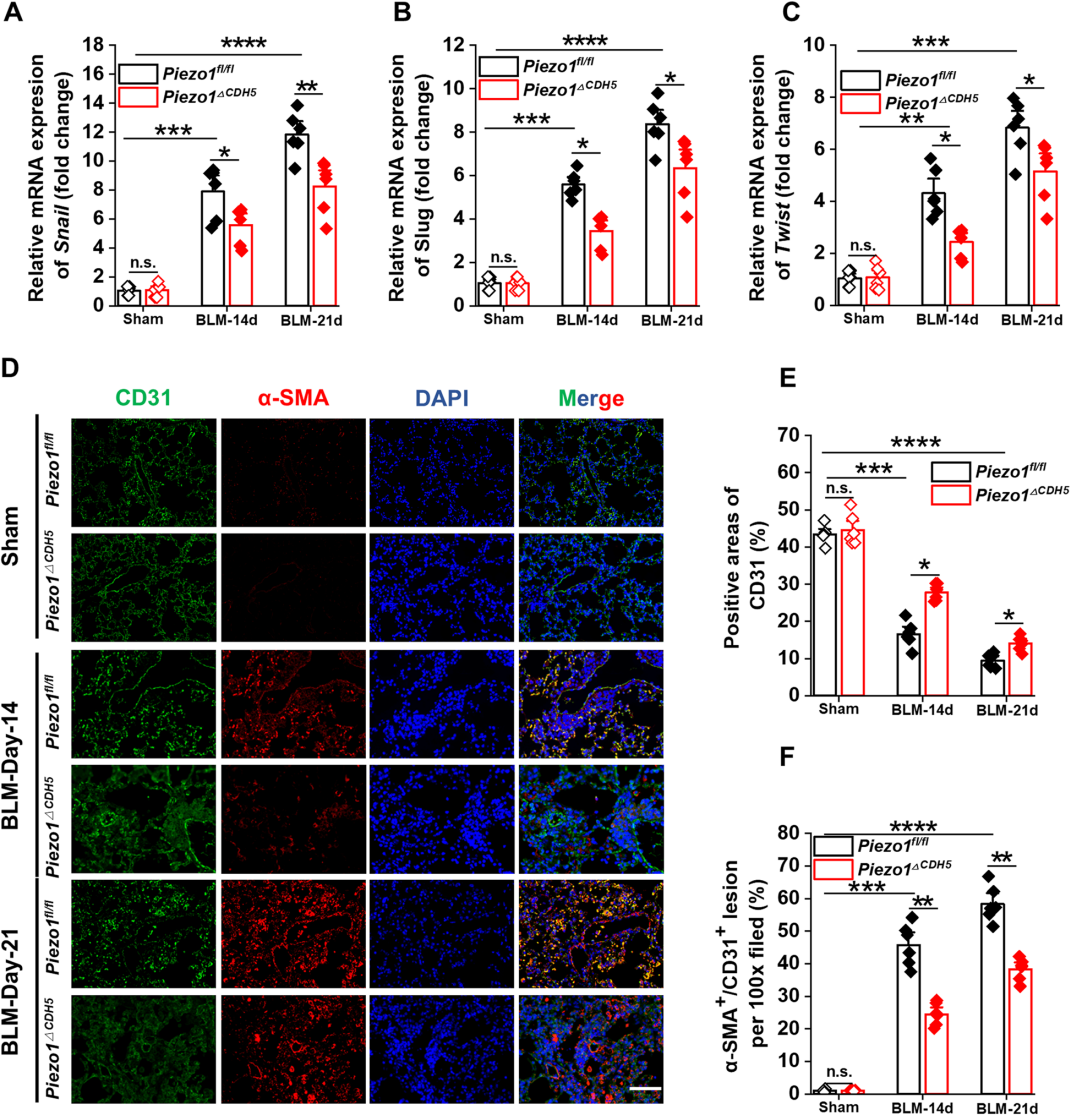
EC-specific Piezo1 deficiency prevent pulmonary fibrosis from EndMT. **A**-**C** Relative mRNA expression of *Snail1*, *Slug* and *Twist* in lung tissues (*n* = 6). **D** Dual immunofluorescent staining targeting on CD31 (green) and α-SMA (red), and** E**–**F** the quantification in lung sections (*n* = 6). Lung tissues and sections were obtained from *Piezo1*^*fl/fl*^ and *Piezo1*^*△CDH5*^ mice subjected to BLM (14/21 days) or sham operation. Data are presented as mean ± SEM. *P < 0.05, **P < 0.01, ***P < 0.001, ****P < 0.0001, ns, not significant

In addition, immunofluorescence, immunohistochemistry, and RT–qPCR consistently showed that the expression levels of FN, COL I, and COL III were also markedly decreased in the lungs of *Piezo1*^*ΔCDH5*^ mice relative to controls (Fig. S3A–O). Collectively, these results indicate that endothelial Piezo1 deficiency effectively suppresses fibroblast activation and excessive extracellular matrix deposition. To further explore the underlying mechanisms, we found that Piezo1 deletion markedly inhibited the expression of transcription factors associated with EndMT and EMT. RT–qPCR analyses demonstrated that the mRNA levels of the key EndMT/EMT-related transcription factors Snail, Slug, and Twist were significantly reduced in the lung tissues of *Piezo1*^*ΔCDH5*^ mice compared with control mice (Fig. 6A–C). Consistently, immunofluorescence staining further confirmed that endothelial Piezo1 deficiency significantly attenuated EndMT and EMT processes in vivo (Fig. 6D–F; Fig. S4A–B). These findings suggest that the amelioration of PF observed in *Piezo1*^*ΔCDH5*^ mice may be associated with suppressed EndMT and a consequent reduction in the secretion of pro-fibrotic mediators. Finally, in wild-type (WT) mice subjected to BLM administration, pharmacological intervention with GsMTx4 produced comparable protective effects, recapitulating the improvements observed in Piezo1-deficient models (Fig. S5A–F).

In summary, endothelial-specific deletion of Piezo1 attenuates BLM-induced PF by inhibiting EndMT, thereby reducing fibroblast activation and collagen-rich extracellular matrix accumulation, and ultimately suppressing fibrotic progression at multiple levels.

### Endothelial Piezo1 deficiency in primary mouse pulmonary endothelial cells suppresses mechanically induced currents

**Fig. 7:**
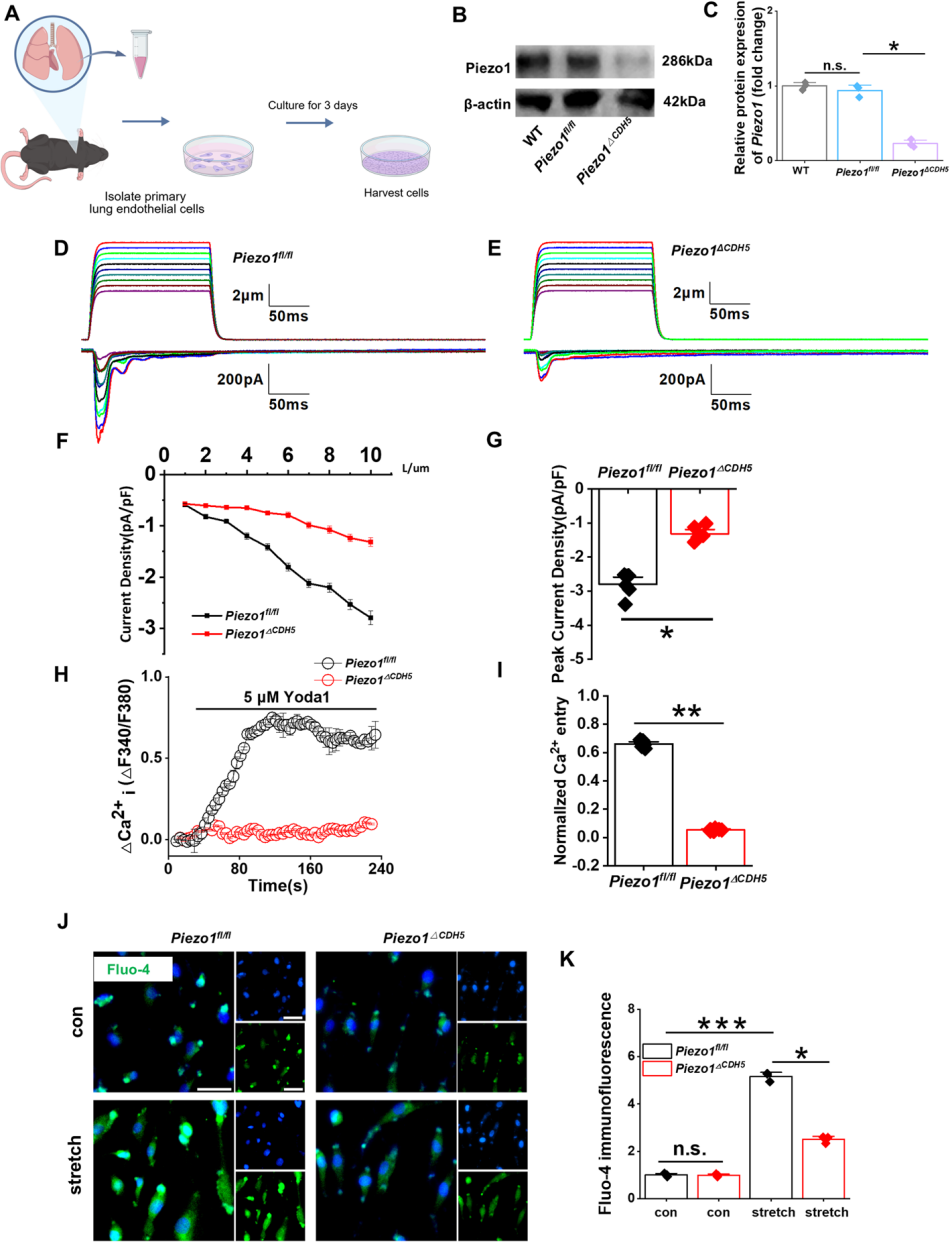
Endothelial Piezo1 knockout inhibits calcium influx stimulated by mechanical stress. **A** Graphic illustration on isolating PMECs. **B** Western blot analysis of the protein expression of Piezo1 in PMECs of each group (*n* = 3). **C** Relative mRNA expression of *Piezo1* in PMECs of each group (*n* = 3). **D** Representative traces of current–pressure relationships (I-μm) for currents evoked by mechanical stimulation on PMECs from *Piezo1*^*fl/fl*^ and **E** *Piezo1*^*△CDH5*^ mice (*n* = 6). **F** Curves and **G** peak current density of each group (*n* = 6). **H** Measurements of intracellular Ca^2+^ flux and **I** peak value in PMECs from *Piezo1*^*fl/fl*^ and *Piezo1*^*△CDH5*^ mice in response to 5 μM Piezo1 specific agonists Yoda1 (*n* = 6). **J** Images and **K** quantification of Ca-imaging by Fluo-4 immunofluorescent staining (*n* = 3). Scale bar, 50 μm. Data are presented as mean ± SEM. *P < 0.05, **P < 0.01, ***P < 0.001, ns, not significant

To determine whether mechanical stretch–induced electrical signal transduction is mediated by Piezo1 activation and to assess whether Piezo1 deficiency disrupts this process, primary mouse pulmonary endothelial cells (PMECs) were isolated from *Piezo1*^*fl/fl*^ and *Piezo1*^*ΔCDH5*^ mice (Fig. 7A). Quantitative PCR and immunoblotting analyses confirmed that Piezo1 expression was significantly reduced in PMECs derived from *Piezo1*^*ΔCDH5*^ mice compared with controls (Fig. 7B–C). Whole-cell patch-clamp recordings demonstrated that in *Piezo1*^*fl/fl*^ PMECs, rapidly activating mechanosensitive inward currents were elicited by mechanical stimulation, with current amplitudes increasing in a stimulus intensity–dependent manner (Fig. 7D; Fig. S6A). In contrast, mechanically evoked currents were markedly diminished in PMECs lacking Piezo1 (Fig. 7E; Fig. S6B). Quantitative analyses further confirmed a significant reduction in mechanically induced currents under Piezo1-deficient conditions (Fig. 7F–G), indicating a positive correlation between mechanical stimulation–evoked currents and Piezo1 expression. These findings demonstrate that Piezo1 deletion in primary mouse pulmonary endothelial cells effectively suppresses mechanically induced ionic currents.

We next evaluated the impact of Piezo1 deficiency on calcium signaling. Upon stimulation with the Piezo1-specific agonist Yoda1 (5 μM), intracellular Ca^2+^ levels rapidly increased in *Piezo1*^*fl/fl*^ PMECs, whereas Ca^2+^ influx was almost completely abolished in *Piezo1*^*ΔCDH5*^ cells (Fig. 7H–I). Consistently, mechanical stretch–induced stimulation followed by Fluo-4–based calcium imaging revealed robust and rapid calcium transients in control cells, which were markedly attenuated in Piezo1-deficient PMECs (Fig. 7J–K). Furthermore, PMECs isolated from fibrotic lungs exhibited characteristic EndMT features, whereas the extent of this phenotypic conversion was significantly reduced in *Piezo1*^*ΔCDH5*^ cells (Fig. S6C–D). These observations suggest that Piezo1 may participate in the regulation of EndMT through modulation of the mechanotransduction–calcium signaling axis.

### Piezo1 regulates EndMT via a calcium signaling Axis

Previous studies have suggested a critical role for mechanosensitive ion channels in fibrotic diseases. In the BLM–induced PF model, the extent of EndMT in PMECs was markedly attenuated in *Piezo1*^*ΔCDH5*^ mice, as evidenced by a significant reduction in CD31^+^/α-SMA^+^ double-positive cells. These observations indicate that BLM-induced PF is closely associated with EndMT and suggest that Piezo1 may participate in this process through a calcium-dependent signaling axis (Fig. S6C). To test this hypothesis, Piezo1 expression was silenced in HUVECs using small interfering RNA (siRNA). Piezo1 knockdown significantly suppressed Yoda1-induced calcium influx (Fig. S7A–B). To establish an in vitro EndMT model, recombinant TGF-β1 was applied as a classical EndMT inducer. Compared with the siControl group, the expression levels of the EndMT-associated transcription factors Snail, Slug, and Twist were markedly reduced in the siPiezo1 group (Fig. S7C–E), accompanied by a pronounced decrease in CD31^+/^α-SMA^+^ co-localized cells (Fig. S7F–G). To further substantiate these findings, the Piezo1 inhibitor GsMTx4 was employed as a pharmacological validation. Western blot and immunofluorescence analyses demonstrated that GsMTx4 effectively suppressed Piezo1 expression (Fig. S8A–D) and significantly attenuated calcium influx (Fig. S8E–F). Compared with TGF-β1 treatment alone, GsMTx4 markedly inhibited the progression of EndMT (Fig. S8G–H). Consistently, RT–qPCR analysis revealed that GsMTx4 significantly reduced TGF-β1–induced mRNA expression of *Col I*, *Col III*, *vimentin*, *α-SMA*, and *TGF-β* itself (Fig. S8I). Western blot analysis further confirmed the inhibitory effects of GsMTx4 on EndMT-related protein expression (Fig. S8J–K). At the morphological level, TGF-β1 stimulation induced a phenotypic transition of endothelial cells from a cobblestone-like morphology to a spindle-shaped, mesenchymal-like appearance, accompanied by a marked enhancement of migratory capacity. In contrast, Piezo1 deficiency effectively prevented these morphological changes and significantly reduced cell migration (Fig. S9A–D).

### Piezo1 regulates EndMT via the Ca^2+^/Calpain–p38/ERK-MAPK–snail signaling axis

**Fig. 8:**
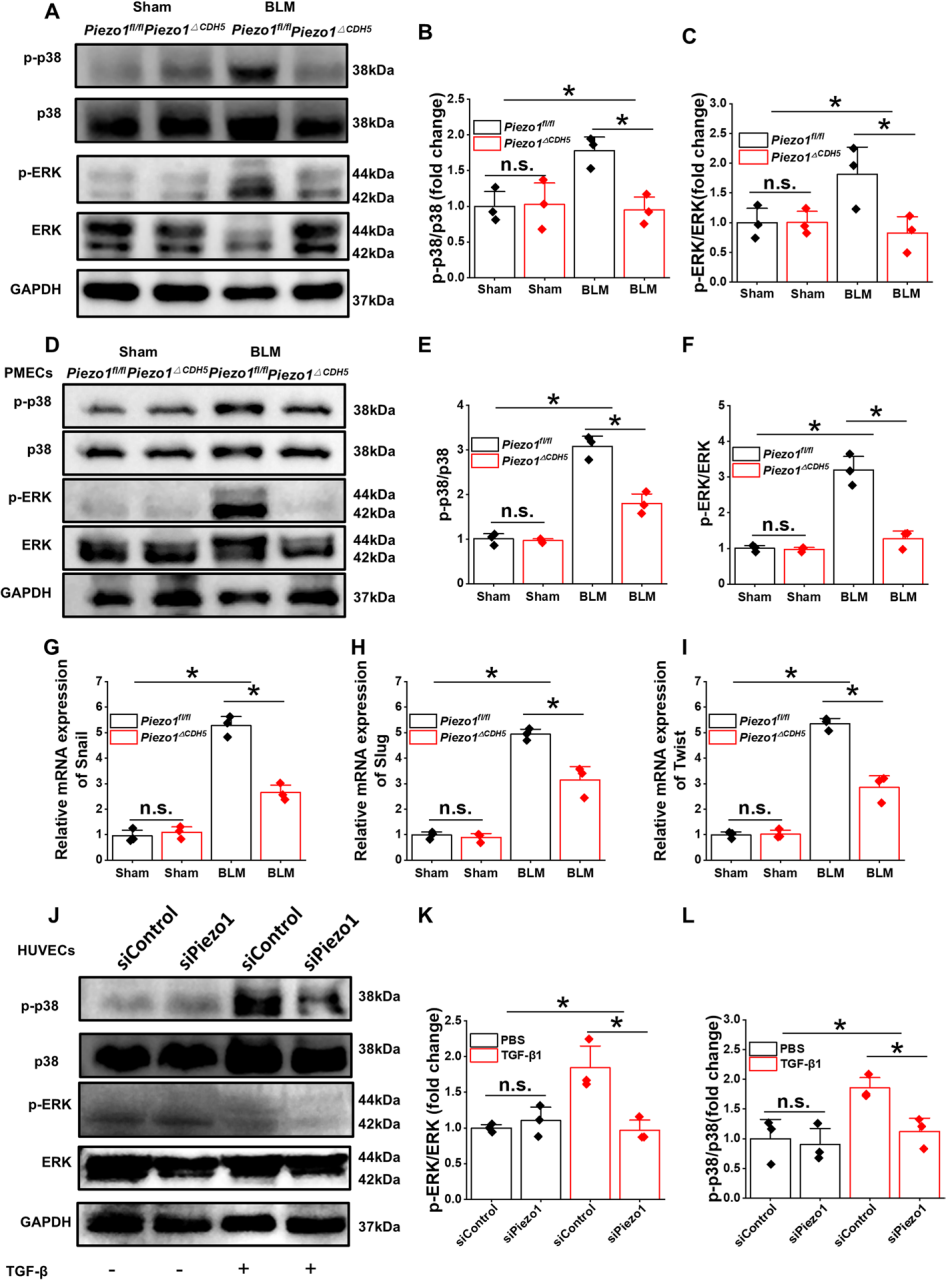
Endothelial *Piezo1* deficiency reduced EndMT via p38-ERK/MAPK axis. **A** Western blot analysis and **B**-**C** quantification of the protein expression of p-p38, p38, p-ERK and ERK in lung tissues from *Piezo1*^*fl/fl*^ and *Piezo1*^Δ*CDH5*^ mice subjected to BLM or sham (*n* = 6). **D** Western blot analysis and **E**–**F** quantification of the protein expression of p-p38, p38, p-ERK and ERK in PMECs from *Piezo1*^*fl/fl*^ and *Piezo1*^Δ*CDH5*^ mice subjected to BLM or sham (*n* = 3). **G**-**I** Relative mRNA expression of *Twist, Snail1* and *Slug* in PMECs from *Piezo1*^*fl/fl*^ and *Piezo1*^Δ*CDH5*^ mice subjected to BLM or sham (*n* = 3). **J** Western blot analysis and **K**-**L** quantification of the protein expression of p-p38, p38, p-ERK and ERK in HUVECs with siControl or si*Piezo1* intervened with PBS or TGF-β1 (*n* = 3). Data are presented as mean ± SEM. *P < 0.05, ns, not significant

Although accumulating evidence suggests a close association between Piezo1 and the EndMT process, the downstream signaling mechanisms underlying this relationship remain incompletely understood. Previous studies have demonstrated that activation of Piezo1 rapidly induces extracellular Ca^2+^ influx, which subsequently modulates the phosphorylation status of the MAPK pathway components p38 and ERK and promotes their nuclear translocation, thereby influencing the activity of transcription factors such as Snail. To investigate the regulatory role of PIEZO1 activation on MAPK signaling, we first analyzed lung tissues from the BLM–induced PF model. The levels of phosphorylated p38 and ERK were significantly elevated in fibrotic lungs compared with control tissues (Fig. 8A–C). In contrast, endothelial-specific deletion of Piezo1 markedly reduced the phosphorylation of p38 and ERK in *Piezo1*^*ΔCDH5*^ mice, while the total protein levels of p38 and ERK remained unchanged. These findings indicate that endothelial Piezo1 deficiency suppresses the activation of MAPK signaling during PF.

Consistently, Western blot analysis of PMECs isolated from fibrotic lungs revealed a pronounced increase in phosphorylated p38 and ERK, whereas PMECs derived from *Piezo1*^*ΔCDH5*^ mice exhibited significantly reduced phosphorylation levels (Fig. 8D–F). In parallel, the mRNA expression of the core EndMT-associated transcription factors *Snail1*, *Slug*, and *Twist* displayed a similar expression pattern (Fig. 8G–I). To further delineate the role of Piezo1 in MAPK activation, endothelial cells were treated with transforming growth factor–β1 (TGF-β1) in vitro. TGF-β1 robustly induced p38 and ERK phosphorylation; however, this effect was markedly attenuated upon Piezo1 knockdown using siRNA, while total p38 and ERK protein levels remained unchanged (Fig. 8J–L). These results suggest that Piezo1 is required for efficient activation of the MAPK signaling cascade in response to profibrotic stimulation.

We next stimulated HUVECs with the Piezo1-specific agonist Yoda1 to assess the role of Piezo1 activation in EndMT. Treatment with Yoda1 alone was insufficient to induce EndMT; however, co-treatment with Yoda1 and TGF-β1 significantly augmented the expression of fibrosis-associated cytokines compared with TGF-β1 treatment alone (Fig. S10A–E). These findings highlight a synergistic role of Piezo1 activation in promoting EndMT under profibrotic conditions.

**Fig. 9:**
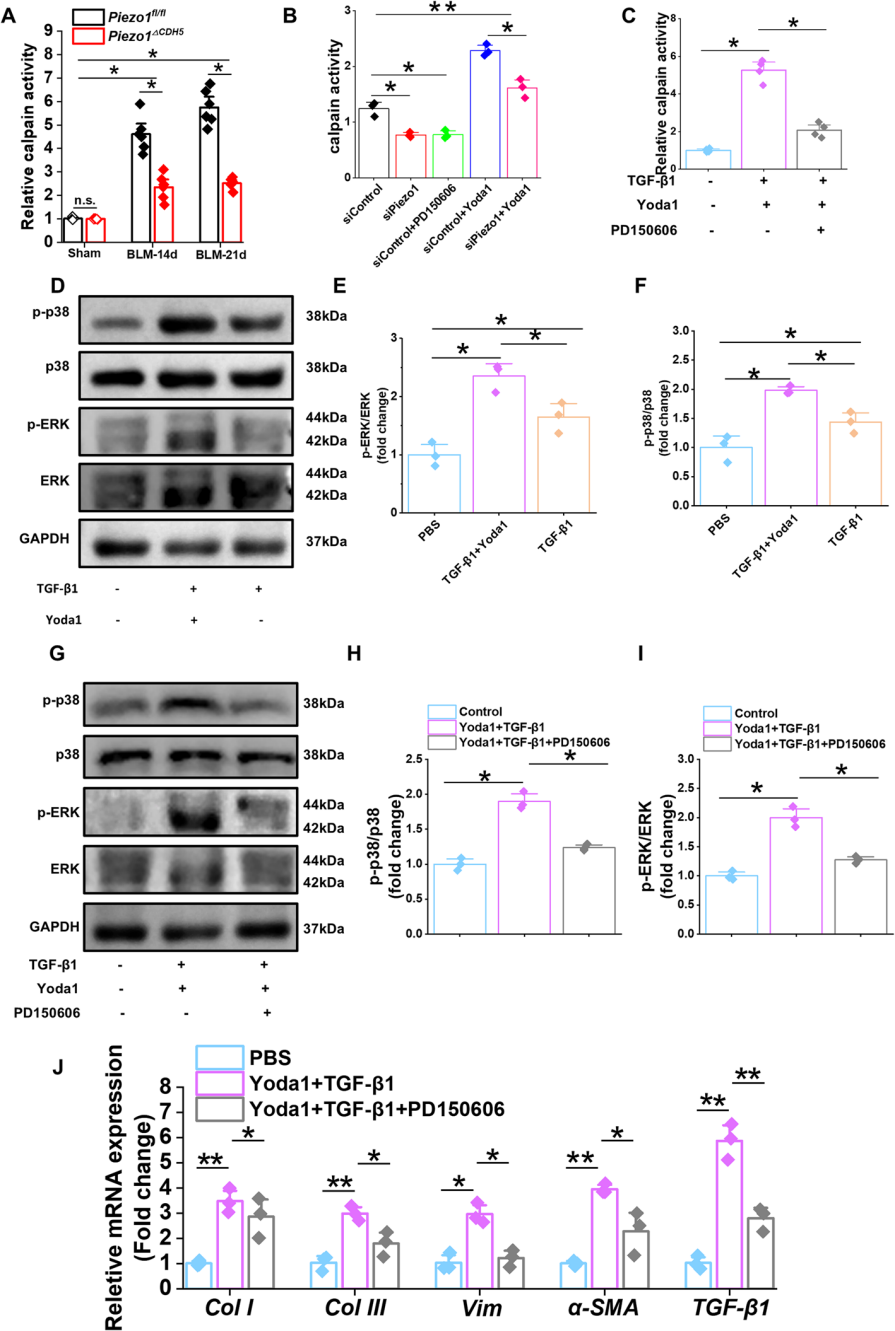
Piezo1 inhibition decreases phosphorylation of p38-ERK/MAPK through Ca^2+^/Calpain. **A** Calpain activity were measured in lung tissue from *Piezo1*^*fl/fl*^ and *Piezo1*^Δ*CDH5*^ mice subjected to BLM or sham (*n* = 6). **B** Yoda 1-induced calpain activity were reduced by calpain inhibitor PD150606 in HUVECs with siControl or si*Piezo1* (*n* = 3). **C** Measurement of calpain activity in HUVECs treated with TGF-β1 and Yoda1 with or without PD150606 (*n* = 3). **D** Western blot analysis and **E**–**F** quantification of the protein expression of p-p38, p38, p-ERK and ERK in HUVECs treated with TGF-β1 with or without Yoda1 (*n* = 3). **G** Western blot analysis and **H**-**I** quantification of the protein expression of p-p38, p38, p-ERK and ERK in HUVECs with TGF-β1 + Yoda1 with or without PD150606 (*n* = 3). **(J)** Relative mRNA expression of *Col I*, *Col III*, *Vim*, *α-SMA* and *TGF-β1* in HUVECs with TGF-β1 and Yoda1 with or without PD150606 (*n* = 3). Data are presented as mean ± SEM. *P < 0.05, **P < 0.01, ns, not significant

Given that Piezo1 activation triggers Ca^2+^ influx and that Ca^2+^ serves as a critical activator of calpain, a Ca^2+^-dependent cysteine protease, we next examined the involvement of calpain in this signaling axis. Calpain activity was significantly reduced in Piezo1-deficient endothelial cells. In the BLM-induced PF model, calpain activity was markedly increased at 14 and 21 days after BLM administration in *Piezo1*^*fl/fl*^ mice, whereas this increase was substantially attenuated in *Piezo1*^*ΔCDH5*^ mice (Fig. 9A), indicating that Piezo1 positively regulates calpain activation during fibrotic progression.

Moreover, Yoda1 stimulation significantly activated calpain in HUVECs, an effect that was effectively suppressed by either Piezo1 knockdown or treatment with the calpain inhibitor PD150606 (Fig. 9B–C). Mechanistically, Yoda1 enhanced TGF-β1–induced phosphorylation of p38 and ERK, whereas this enhancement was reversed by PD150606 (Fig. 9D–I). Similar results were obtained in isolated PMECs (Fig. S10F–K). Finally, pharmacological inhibition of calpain with PD150606 significantly reduced Yoda1/TGF-β1–induced calpain activity and attenuated EndMT by downregulating the mRNA expression of *Col I*, *Col III*, *vimentin*, and *α-SMA* (Fig. 9J).

**Fig. 10:**
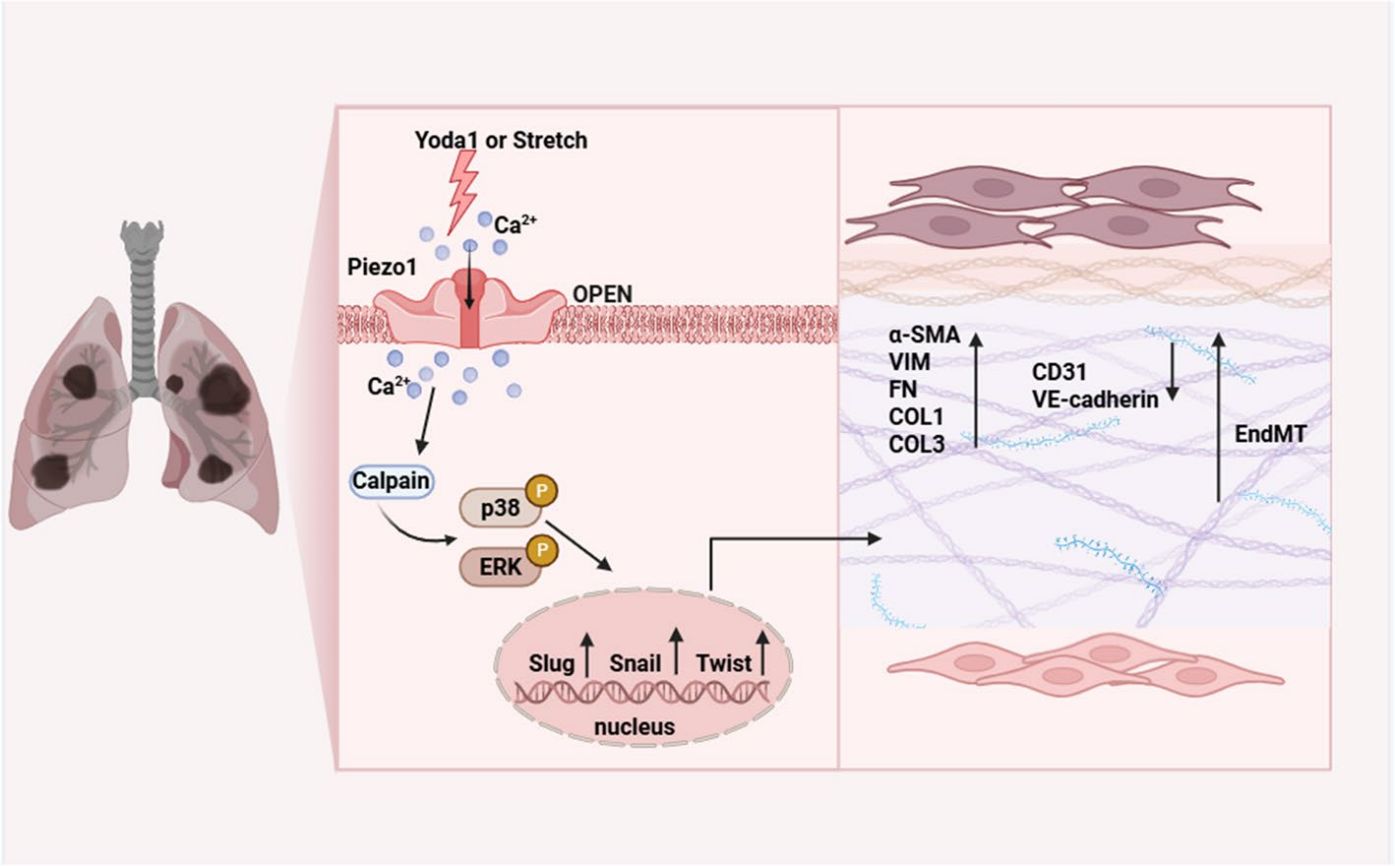
Graphic illustration on Piezo1 activation modulates the progression of pulmonary fibrosis by regulating EndMT via the Ca^2+^/calpain/p38/ERK-MAPK axis

Collectively, these results demonstrate that Piezo1 promotes EndMT by mediating Ca^2+^ influx and subsequent calpain activation, which in turn triggers the p38/ERK-MAPK signaling cascade. Together, these findings define a Piezo1–Ca^2+^/Calpain–MAPK signaling axis that critically regulates EndMT during PF (Fig. 10).

## Discussion

PF represents the terminal stage of severe pulmonary diseases, with current therapeutic strategies primarily relying on antifibrotic agents to slow disease progression [1–3]. However, the underlying pathophysiological mechanisms remain incompletely elucidated, thereby limiting the development of effective treatment strategies. This study identifies a critical role of endothelial Piezo1 in the pathogenesis and progression of PF. First, we observed a significant upregulation of Piezo1 expression in the lung tissues of a murine model of PF. Furthermore, *Piezo1*^*ΔCDH5*^ markedly attenuated PF by reducing the formation of fibrotic lesions. The loss of Piezo1 inhibited aberrant calcium influx and suppressed calpain activation [36], thereby mitigating EndMT and ultimately alleviating PF [37, 38]. In addition, endothelial Piezo1 deletion reduced macrophage recruitment and significantly suppressed inflammatory responses, immune cell infiltration and apoptosis.

Beyond PF, Piezo1 also plays a critical role in fibrosis occurring in other tissues. For instance, in cardiac fibroblasts, Piezo1 mediates cardiac remodeling by activating calcium ion influx and reactive oxygen species (ROS) signaling [33, 34]. Additionally, in myeloid cells, Piezo1 accelerates the progression of renal fibrosis by promoting intrinsic inflammatory processes [29]. These findings further support the broad involvement of Piezo1 in fibrotic pathophysiology and suggest that it may serve as a potential therapeutic target across multiple fibrotic diseases.

The present study further investigates the mechanistic role of Piezo1 in lung injury and fibrosis. During PF, pulmonary vascular pressure is markedly elevated, and the tension within the lung parenchyma increases, leading to alterations in the endothelial microenvironment and subsequently enhancing cellular membrane tension [39, 40]. This mechanical stress modulates cellular functions through Piezo1-mediated Ca^2+^ signaling pathways [41]. As a mechanosensitive cation channel, Piezo1 is capable of transducing mechanical stimuli into biochemical signals. Previous studies have demonstrated that Piezo1 expression is significantly upregulated in endothelial cells following pulmonary arterial hypertension (PAH), with even further elevation observed during PF [42]. Notably, the loss of endothelial Piezo1 has been shown to mitigate pulmonary fibrotic injury, highlighting Piezo1 as a key regulatory factor in this pathological process. Ca^2+^ homeostasis is essential for the maintenance of cellular physiological functions [36]. Ca^2+^ acts as an allosteric effector, binding to calpain and inducing conformational changes that relieve autoinhibition, thereby activating its proteolytic function. This process is one of the key pathways by which cells respond to Ca^2+^ signals [43]. During the progression of PF, calpain activity is significantly enhanced. As a downstream target of Ca^2+^, calpain is activated when intracellular Ca^2+^ levels rise [44]. Notably, the absence of Piezo1 inhibits this process. Previous studies have demonstrated a functional coupling between Piezo1 and the Ca^2+^/calpain signaling pathway. Inhibition of calpain activity has been shown to reduce EndMT and decrease endothelial cell migration capacity. In the present cellular experiments, we observed a significant increase in calpain activity under TGF-β1-induced conditions, which was reversed upon Piezo1 inhibition, further confirming the upstream regulatory role of Piezo1 in calpain activation [28, 29, 45–47]. EndMT is a common feature in all fibrotic diseases and plays a crucial role in disease progression [48–50].

The MAPK pathway is fundamentally involved in numerous physiological activities, such as cell death (apoptosis), growth (proliferation), specialization (differentiation), and the development of fibrotic tissue [51, 52]. Our study found a significant increase in EndMT in a PF model, accompanied by elevated phosphorylation levels of p38 and ERK MAPK pathways [53–55]. However, *Piezo1*^*△CDH5*^ reversed the upregulation of these phosphorylation levels and reduced the expression of EndMT-associated transcription factors, including *Slug, Snail, and Twist* [56–58]. These results suggest that Piezo1 may mediate EndMT through the regulation of the MAPK signaling pathway. In vitro cellular experiments revealed a close association between TGF-β1-induced EndMT and calpain activation. This phenomenon was significantly inhibited following treatment with GsMTx4 or *Piezo1*-siRNA [44]. Moreover, the calpain inhibitor PD150606 notably inhibited both Yoda1- and TGF-β1-induced EndMT and reduced the phosphorylation levels of p38 and ERK MAPK pathways. These findings further validate calpain as a downstream effector of Piezo1 and highlight the crucial regulatory role of the Ca^2+^/calpain signaling pathway in the EndMT process. To further confirm these mechanisms, we established a tamoxifen-induced conditional *Piezo1* knockout mouse model (*Piezo1*^*△CDH5*^) [59]. Experimental results indicated that inhibition of EndMT reduced the extent of PF lesions and suppressed inflammatory. While this study elucidates the functional importance of the Ca^2+^/calpain/MAPK axis, it is imperative to acknowledge its limitations. The downstream signaling network of Piezo1 is notably complex. Beyond the pathway delineated herein, extant literature demonstrates that alternative signaling cascades, including PI3K/Akt, RhoA/ROCK, and the canonical TGF-β/Smad pathway, are also integrally involved in the regulation of pulmonary fibrosis and EndMT [60–62]. Building upon the present findings, future investigations are warranted to dissect the potential crosstalk and hierarchical relationships between Piezo1 and these alternative pathways (e.g., PI3K/Akt, RhoA/ROCK) to construct a more comprehensive and integrated regulatory network. In conclusion, this study elucidates the key role of endothelial Piezo1 in regulating EndMT via the Ca^2^^+^/calpain-p38/ERK-MAPK pathway during the progression of PF. These results enhance our comprehension of the underlying mechanisms driving PF and offer a theoretical foundation for designing antifibrotic therapies targeting Piezo1. Targeted interventions against Piezo1 may offer novel therapeutic approaches for mechanical stress-related lung injury.

## Supplementary Information

Supplementary Material 1: Table S2. Sequences of the primers used for RT-qPCR. Table S2. List of antibodies. Figure S1. Validation of inducible endothelial-specific *Piezo1* knockout mice. (A) Genotyping of *Piezo1*-*Cdh5*-Cre^+^ (*Piezo1*^Δ*CDH5*^) mice with *Piezo1*-*Cdh5*-Cre^−^ (*Piezo1*^*fl/fl*^) mice as controls (*n* = 3). (B) Schema demonstrating the process of *Piezo1*^Δ*CDH5*^ mice using tamoxifen-inducible *Cdh5*-promoter driven CreER^T2^. (C) Relative mRNA expression of *Piezo1,* (D) example traces and (E) quantification of Yoda1-induced calcium influx assays in isolated liver endothelial cells from *Piezo1*^Δ*CDH5*^ and *Piezo1*^*fl/fl*^ mice were validated for *Piezo1* knockout (*n* = 6). Data are presented as mean ± SEM. ***P < 0.001. Figure S2. EC-specific *Piezo1* knockout decreases inflammatory cell infiltration and inflammatory cytokines level in pulmonary fibrosis. (A) Images and (B) quantitative evaluation of immunofluorescent staining targeting F4/80 (*n* = 6). Scale bar, 50 μm. (C) Images and (D) quantitative evaluation of immunohistochemistry staining targeting F4/80 (*n* = 6). Scale bar, 50 μm. (E–G) Relative mRNA expression of *TNF-α, IL-6*, and *IL-1β* in lung tissues (*n* = 6). (H-J) Quantitative analysis of TNF-α, IL-6, and IL-1β in BALF of each group by ELISA (*n* = 6). (K-L) Cell number of neutrophils and macrophages are counted in BALF of each group (*n* = 6). The BALF and lung sections were obtained from *Piezo1*^*fl/fl*^ and *Piezo1*^*△CDH5*^ mice subjected to BLM (14/21 days) or sham operation. Data are presented as mean ± SEM. *P < 0.05, **P < 0.01, ***P < 0.001, ****P < 0.0001, ns, not significant. Figure S3. Endothelial Piezo1 deficiency mitigates pulmonary fibrosis. (A) Images and (B) quantification of immunohistochemistry staining with fibronectin in lung sections (*n* = 6). Scale bar, 50 μm. (C) Images and (D) quantification of immunofluorescent staining with fibronectin in lung sections (*n* = 6). Scale bar, 50 μm. (E) Relative mRNA expression of *fibronectin* in lung tissues (*n* = 6). (F) Images and (G) quantification of immunohistochemistry staining with Col I in lung sections (*n* = 6). Scale bar, 50 μm. (H) Images and (I) quantification of immunofluorescent staining with Col I in lung sections (*n* = 6). Scale bar, 50 μm. (J) Relative mRNA expression of *Col I* in lung tissues (*n* = 6). (K) Images and (L) quantification of immunohistochemistry staining with collagen III in lung sections (*n* = 6). Scale bar, 50 μm. (M) Images and (N) quantification of immunofluorescent staining with Col III in lung sections (*n* = 6). Scale bar, 50 μm. (O) Relative mRNA expression of *Col III* in lung tissues (*n* = 6). Lung tissues and sections were obtained from *Piezo1*^*fl/fl*^ and *Piezo1*^*△CDH5*^ mice subjected to BLM (14/21 days) or sham operation. Data are presented as mean ± SEM. *P < 0.05, **P < 0.01, ***P < 0.001, ns, not significant. Figure S4. EC-specific Piezo1 knockout decreases pulmonary fibrosis by inhibiting EMT. (A) Dual immunofluorescent staining targeting on E-cadherin (green) and α-SMA (red), and (B) the quantification in lung sections from *Piezo1*^*fl/fl*^ and *Piezo1*^*△CDH5*^ mice subjected to BLM (14/21 days) or sham operation (*n* = 6). Scale bar, 50 μm. Data are presented as mean ± SEM. *P < 0.05, **P < 0.01, ***P < 0.001, ****P < 0.0001, ns, not significant. Figure S5. Inhibition of Piezo1 exerts protective effects on pulmonary fibrosis. (A) H&E staining images and (B) lung injury score of each group (*n* = 6). Scale bar, 50 μm. (C) Images and (D) quantification of collagen positive areas with Sirius red staining (*n* = 6). Scale bar, 50 μm. (E) Masson’s trichrome staining images and (F) Ashcroft score (*n* = 6). Scale bar, 50 μm. Data are presented as mean ± SEM. *P < 0.05, **P < 0.01, ***P < 0.001, ****P < 0.0001, ns, not significant. Figure S6. EMT reduced in PMECs isolated from Piezo1-ECKO mice. (A) Representative traces of current–pressure relationships (I-μm) for currents evoked by mechanical stimulation on PMECs from *Piezo1*^*fl/fl*^ and (B) *Piezo1*^*△CDH5*^ mice (*n* = 6). (C) Dual immunofluorescent staining targeting on CD31 (green) and α-SMA (red), and (D) the quantification in PMECs (*n* = 6). Scale bar, 50 μm. Data are presented as mean ± SEM. *P < 0.05, **P < 0.01, ns, not significant. Figure S7. Piezo1 knockdown alleviates EndMT in vitro. (A) Measurements of intracellular Ca^2+^ flux and (B) peak value in HUVECs with siControl or si*Piezo1* in response to 5 μM Yoda1 (*n* = 8). (C-E) Relative mRNA expression of *Twist*, *Snail1* and *Slug* in HUVECs with siControl or si*Piezo1* treated with PBS or TGF-β1 (*n* = 6). (F) Dual immunofluorescent staining targeting on CD31 (green) and α-SMA (red), and (G) the quantification in HUVECs with siControl or si*Piezo1* treated with PBS or TGF-β1 (*n* = 6). Scale bar, 50 μm. Data are presented as mean ± SEM. *P < 0.05, **P < 0.01, ns, not significant. Figure S8. Piezo1 inhibitor GsMTx4 decreases EndMT induced by TGF-β1 in vitro. HUVECs were treated by PBS or TGF-β1 or TGF-β1 + GsMTx4. (A) Western blot analysis and (B) quantification of the protein expression of Piezo1 in HUVECs of each group (*n* = 3). (C) Images and (D) quantification of immunofluorescent staining with Piezo1 in HUVECs of each group (*n* = 6). (E–F) GsMTx4 inhibited the intracellular Ca^2+^ flux induced by Piezo1 activation in HUVECs in response to 5 μM Yoda1 (*n* = 6). (G) Images and (H) quantification of dual immunofluorescence staining with VE (green) and α-SMA (red) in HUVECs of each group (*n* = 3). Scale bar, 50 μm. (I) Relative mRNA expression of *Piezo1*, *Col I*, *Col III*, *Vim*, *FN*, *α-SMA* and *TGF-β1* in HUVECs of each group (*n* = 6). (J) Western blot analysis and (K) quantitative data of CD31, VE-cad, α-SMA and Vim expression in HUVECs treated by PBS or TGF-β1 or TGF-β1 + GsMTx4 (*n* = 3). Data are presented as mean ± SEM.; *P < 0.05. Figure S9. Endothelial *Piezo1* knockdown inhibited EndMT. HUVECs were treated by PBS or TGF-β1 or TGF-β1 + GsMTx4. (A) Images and (B) quantitative of immunofluorescence staining with CD31, VE, α-SMA, TGF-β1 and Vim in HUVECs of each group (*n* = 3). Scale bar, 50 μm. (C) Images of migration assay and (D) percentage of migration in 24 h were measured in HUVECs of each group (*n* = 3). Data are presented as mean ± SEM. *P < 0.05. Figure S10. Piezo1 inhibition decreases phosphorylation of p38-ERK/MAPK through Ca^2+^/Calpain. (A-E) Relative mRNA expression of *Col I*, *Col III*, *Vim*, *α-SMA* and *TGF-β* in HUVECs treated with or without TGF-β1 and Yoda1 (*n* = 3). (F) Western blot analysis and (G-H) quantification of the protein expression of p-p38, p38, p-ERK and ERK in PMECs treated with TGF-β1 with or without Yoda1 (*n* = 3). (I) Western blot analysis and (J-K) quantification of the protein expression of p-p38, p38, p-ERK and ERK in PMECs with TGF-β1 + Yoda1 with or without PD150606 (*n* = 3). Data are presented as mean ± SEM. *P < 0.05.

Supplementary Material 2.

## Data Availability

All data are available from the corresponding authors upon reasonable request.

## Abbreviations

BALF: Bronchoalveolar lavage fluid.
BLM: Bleomycin
Ca^2+^: Calcium ion
EC: Endothelial cell
ECM: Extracellular matrix
ELISA: Enzyme-linked immunosorbent assay
EMT: Epithelial–mesenchymal transition
EndMT: Endothelial–mesenchymal transition
ILDs: Interstitial lung diseases
IPF: Idiopathic pulmonary fibrosis
IL-1β: Interleukin-1β
IL-6: Interleukin-6
PF: Pulmonary fibrosis
PAH: Pulmonary arterial hypertension
PMECs: Primary mouse pulmonary endothelial cells
siRNA: Small interfering RNA
TNF-α: Tumor necrosis factor–α
