# Supplementary material for: Piezo1 specific deletion in endothelial cell protects the progression of pulmonary fibrosis in mice
Source: Cell Commun Signal. 2026 Feb 28;24:207. doi: 10.1186/s12964-026-02758-7 (PMC13049882; doi:10.1186/s12964-026-02758-7)
Supplement: Supplementary file 1 — Supplementary Material 1: Table S2. Sequences of the primers used for RT-qPCR. Table S2. List of antibodies. Figure S1. Validation of inducible endothelial-specific Piezo1 knockout mice. (A) Genotyping of Piezo1-Cdh5-Cre+ (Piezo1ΔCDH5) mice with Piezo1-Cdh5-Cre− (Piezo1fl/fl) mice as controls (n = 3). (B) Schema demonstrating the process of Piezo1ΔCDH5 mice using tamoxifen-inducible Cdh5-promoter driven CreERT2. (C) Relative mRNA expression of Piezo1, (D) example traces and (E) quantification of Yoda1-induced calcium influx assays in isolated liver endothelial cells from Piezo1ΔCDH5 and Piezo1fl/fl mice were validated for Piezo1 knockout (n = 6). Data are presented as mean ± SEM. ***P < 0.001. Figure S2. EC-specific Piezo1 knockout decreases inflammatory cell infiltration and inflammatory cytokines level in pulmonary fibrosis. (A) Images and (B) quantitative evaluation of immunofluorescent staining targeting F4/80 (n = 6). Scale bar, 50 μm. (C) Images and (D) quantitative evaluation of immunohistochemistry staining targeting F4/80 (n = 6). Scale bar, 50 μm. (E–G) Relative mRNA expression of TNF-α, IL-6, and IL-1β in lung tissues (n = 6). (H-J) Quantitative analysis of TNF-α, IL-6, and IL-1β in BALF of each group by ELISA (n = 6). (K-L) Cell number of neutrophils and macrophages are counted in BALF of each group (n = 6). The BALF and lung sections were obtained from Piezo1fl/fl and Piezo1△CDH5 mice subjected to BLM (14/21 days) or sham operation. Data are presented as mean ± SEM. *P < 0.05, **P < 0.01, ***P < 0.001, ****P < 0.0001, ns, not significant. Figure S3. Endothelial Piezo1 deficiency mitigates pulmonary fibrosis. (A) Images and (B) quantification of immunohistochemistry staining with fibronectin in lung sections (n = 6). Scale bar, 50 μm. (C) Images and (D) quantification of immunofluorescent staining with fibronectin in lung sections (n = 6). Scale bar, 50 μm. (E) Relative mRNA expression of fibronectin in lung tissues (n = 6). (F) Images and (G) quantifica [file 12964_2026_2758_MOESM1_ESM.docx]

**Table S1. Sequences of the primers used for RT-qPCR.**

| **Gene name** | **Species** | **Forward primers** | **Reverse primers** |
| --- | --- | --- | --- |
| *18S* | Mouse/human | TGGTTGCAAAGCTGAAACTTAAAG | AGTCAAATTAAGCCGCAGGC |
| *Piezo1* | Mouse | CACAAAGTACCGGGCG | AAAGTAAATGCACTTGACG |
| *Collagen I* | Mouse | GCTCCTCTTAGGGGCCACT | ATTGGGGACCCTTAGGCCAT |
| *Collagen III* | Mouse | CCACGAGGTGACAAAGGTGA | GCCAGGGAATCCTCGATGT |
| *Vimentin* | Mouse | CAGAGAGAGGAAGCCGAAAG | ATGCTGTTCCTGAATCTGGG |
| *Fibronectin* | Mouse | ATGTGGACCCCTCCTGATAGT | GCCCAGTGATTTCAGCAAAGG |
| *α-SMA* | Mouse | CCCAGACATCAGGGAGTAATGG | TCTATCGGATACTTCAGCGTCA |
| *TGF-β* | Mouse | GAGCCCGAAGCGGACTACTA | TGGTTTTCTCATAGATGGCGTT |
| *TNF-α* | Mouse | ACGGCATGGATCTCAAAGAC | CGGACTCCGCAAAGTCTAAG |
| *IL1β* | Mouse | AGCTCTCCACCTCAATGGAC | GACAGGCTTGTGCTCTGCTT |
| *IL6* | Mouse | TCCATCCAGTTGCCTTCTTG | GGTCTGTTGGGAGTGGTATC |
| *IL10* | Mouse | AGCCTTATCGGAAATGATCCAGT | GGCCTTGTAGACACCTTGGT |
| *Snail1* | Mouse | CGAGTGGTTCTTCTGCGCTA | CTGCTGGAAGGTAAACTCTGGA |
| *Slug* | Mouse | CTGGGCTGGCCAAACACAA | GCTCACATATTCCTTGTCACAGAAC |
| *Twist* | Mouse | GCCGGAGACCTAGATGTCATT | TTTTAAAAGTGCGCCCCACG |
| *Collagen I* | Human | CCCGGGTTTCAGAGACAACTTC | TCCACATGCTTTATTCCAGCAATC |
| *Collagen III* | Human | GGAGCTGGCTACTTCTCGC | GGGAACATCCTCCTTCAACAG |
| *Vimentin* | Human | CTTCCAAACTTTTCCTCCC | AGTTTCGTTGATAACCTGTCC |
| *α-SMA* | Human | GGTGCTGTCTCTCTATGCCT | AAGGAATAGCCACGCTCAGT |
| *TGF-β* | Human | ACAGCAACAATTCCTGGCGA | CCGTTGATGTCCACTTGCAG |
| *Snail1* | Human | TCTAGGCCCTGGCTGCTACAA | ACATCTGAGTGGGTCTGGAGGTG |
| *Slug* | Human | AGCAGTTGCACTGTGATGCC | ACACAGCAGCCAGATTCCTC |
| *Twist* | Human | GTCCGCAGTCTTACGAGGAG | GCTTGAGGGTCTGAATCTTGCT |

**Table S2. List of antibodies**

| **Antibodies** | **Origin** | **Product code** | **Dilution** | **Application** |
| --- | --- | --- | --- | --- |
| Anti-Piezo1 antibody | Proteintech | 15939-1-AP | 1:500 | IHC |
| Anti-Piezo1 antibody | Proteintech | 15939-1-AP | 1:200 | IF |
| Anti-CollagenI antibody | Servicebio | GB11022-3 | 1:200 | IHC, IF |
| Anti-CollagenIII antibody | Servicebio | GB111629 | 1:200 | IHC, IF |
| Anti-Vimentin antibody | Abcam | ab92547 | 1:200 | IHC, IF |
| Anti-Fibronectin antibody | Abcam | ab2413 | 1:200 | IHC, IF |
| Anti-α-SMA antibody | Abcam | ab5694 | 1:400 | IHC, IF |
| Anti-F4/80 antibody | Servicebio | GB113373 | 1:200 | IHC, IF |
| Anti-CD31 antibody | Proteintech | 28083-1-AP | 1:400 | IHC, IF |
| Anti-Vimentin antibody | Proteintech | 10366-1-AP | 1:400 | IHC, IF |
| Anti-α-SMA antibody | Proteintech | 67735-1-Ig | 1:400 | IHC, IF |
| Anti-TGF beta 1 antibody | Proteintech | 26155-1-AP | 1:200 | IHC, IF |
| Anti-CD31 antibody | Abcam | ab222783 | 1:100 | IHC, IF |
| Anti-VE Cadherin antibody | Abcam | ab33168 | 1:200 | IHC, IF |
| HRP anti-rabbit IgG | Servicebio | G1213 | 1:200 | IHC |
| HRP anti-mouse IgG | Servicebio | G1214 | 1:200 | IHC |
| Alexa Fluor™594 goat anti-rabbit IgG (H+L) | Invitrogen | A11012 | 1:800 | IF |
| Alexa Fluor™488 goat anti-rabbit IgG (H+L) | Invitrogen | A11008 | 1:800 | IF |
| Alexa Fluor™488 goat anti-rat IgG (H+L) | Invitrogen | A11006 | 1:800 | IF |
| Goat Anti-RatIgG/S Alexa Fluor 647 | Solarbio | K0032G | 1:800 | IF |
| Anti-CD31 antibody | Proteintech | 28083-1-AP | 1:1000 | WB |
| Anti-Vimentin antibody | Proteintech | 10366-1-AP | 1:2000 | WB |
| Anti-α-SMA antibody | Proteintech | 67735-1-Ig | 1:2000 | WB |
| Anti-CD31 antibody | Abcam | ab222783 | 1:2000 | WB |
| Anti-VE Cadherin antibody | Abcam | ab33168 | 1:1500 | WB |
| Anti-TGF beta 1 antibody | Proteintech | 26155-1-AP | 1:2000 | WB |
| Anti-TGF beta 1 antibody | CST | 3711 | 1:1000 | WB |
| Anti-p38 MAPK antibody | CST | 8690T | 1:1000 | WB |
| Anti-P-p38 MAPK antibody | CST | 4511T | 1:1000 | WB |
| Anti-p44/42 MAPK (ERK 1/2) antibody | CST | 4695T | 1:1000 | WB |
| Anti-P-p44/42 MAPK (ERK 1/2) antibody | CST | 4370T | 1:1000 | WB |
| GAPDH | CST | 2118 | 1:2000 | WB |
| β-tubulin | CST | 2128 | 1:1000 | WB |
| Beta Actin Polyclonal antibody | Proteintech | 20536-1-AP | 1:3000 | WB |
| HRP-conjugated anti-rabbit IgG | CST | 7074s | 1:3000 | WB |
| HRP-conjugated anti-mouse IgG | CST | 7076s | 1:3000 | WB |


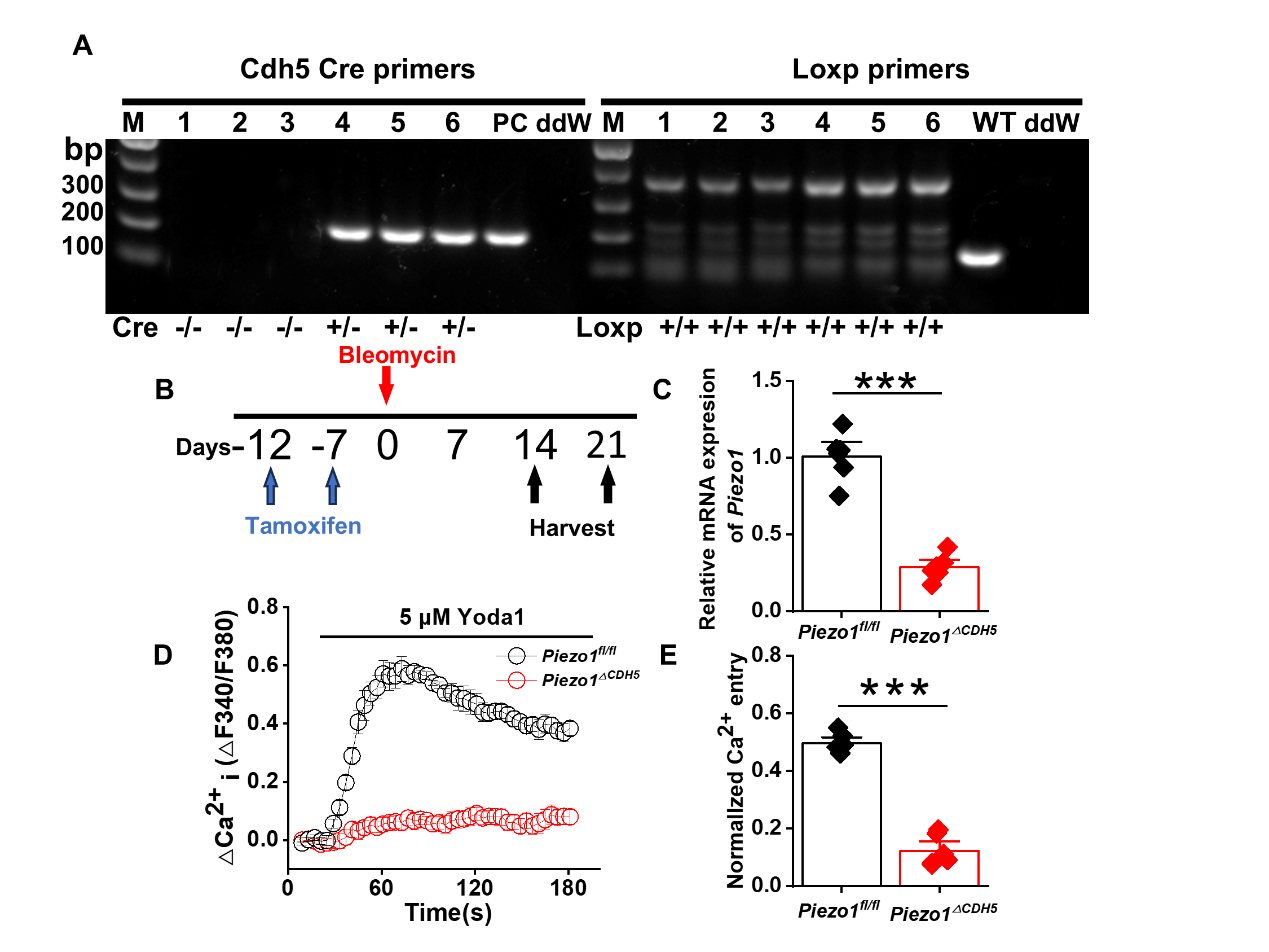


**Figure S1. Validation of inducible endothelial-specific *Piezo1* knockout mice. (A)** Genotyping of *Piezo1*-*Cdh5*-Cre^+^ (*Piezo1*^Δ^*^CDH5^*) mice with *Piezo1*-*Cdh5*-Cre^-^ (*Piezo1^fl/fl^*) mice as controls (n=3). **(B)** Schema demonstrating the process of *Piezo1*^Δ^*^CDH5^* mice using tamoxifen-inducible *Cdh5*-promoter driven CreER^T2^. **(C)** Relative mRNA expression of *Piezo1,* **(D)** example traces and **(E)** quantification of Yoda1-induced calcium influx assays in isolated liver endothelial cells from *Piezo1*^Δ^*^CDH5^* and *Piezo1^fl/fl^* mice were validated for *Piezo1* knockout (n=6). Data are presented as mean ± SEM. ***P < 0.001.


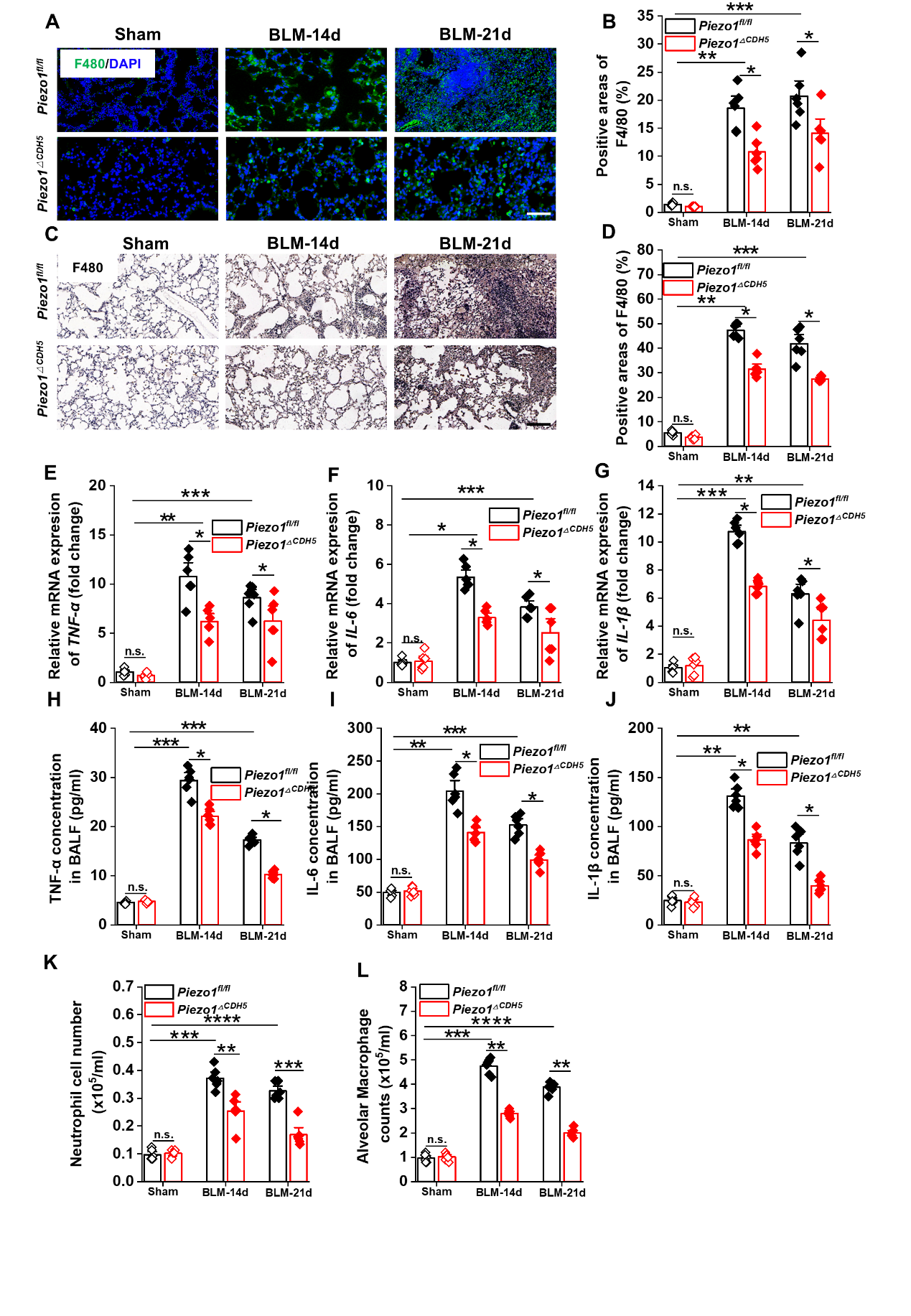


**Figure S2. EC-specific *Piezo1* knockout decreases inflammatory cell infiltration and inflammatory cytokines level in pulmonary fibrosis. (A)** Images and **(B)** quantitative evaluation of immunofluorescent staining targeting F4/80 (n=6). Scale bar, 50 μm. **(C)** Images and **(D)** quantitative evaluation of immunohistochemistry staining targeting F4/80 (n=6). Scale bar, 50 μm. **(E**-**G)** Relative mRNA expression of *TNF-α, IL-6*, and *IL-1β* in lung tissues (n=6). **(H-J)** Quantitative analysis of TNF-α, IL-6, and IL-1β in BALF of each group by ELISA (n=6). **(K-L)** Cell number of neutrophils and macrophages are counted in BALF of each group (n=6). The BALF and lung sections were obtained from *Piezo1^fl/fl^* and *Piezo1^△CDH5^* mice subjected to BLM (14/21 days) or sham operation. Data are presented as mean ± SEM. *P<0.05, **P<0.01, ***P < 0.001, ****P < 0.0001, ns, not significant.


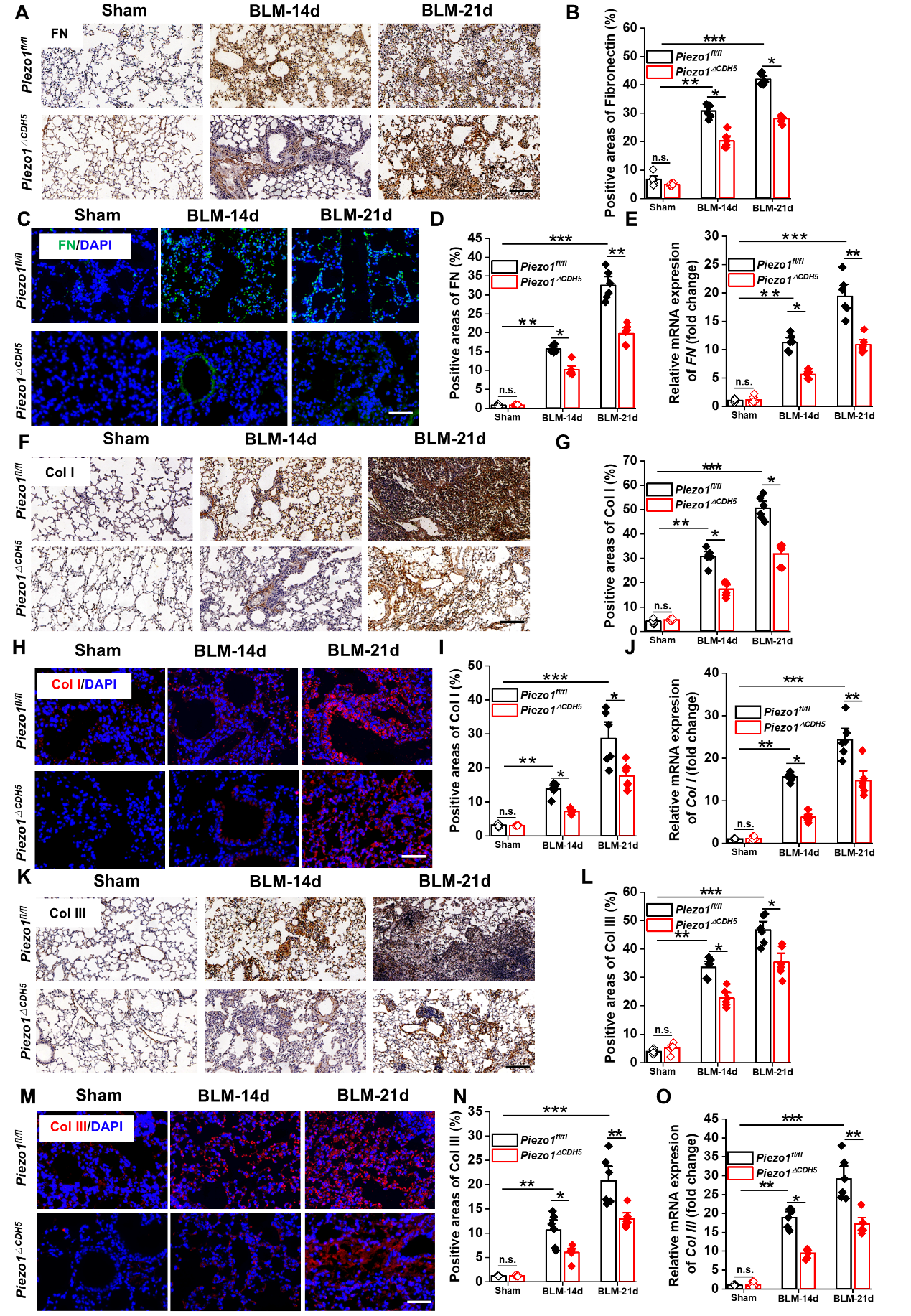


**Figure S3. Endothelial Piezo1 deficiency mitigates pulmonary fibrosis.** **(A)** Images and **(B)** quantification of immunohistochemistry staining with fibronectin in lung sections (n=6). Scale bar, 50 μm. **(C)** Images and **(D)** quantification of immunofluorescent staining with fibronectin in lung sections (n=6). Scale bar, 50 μm. **(E)** Relative mRNA expression of *fibronectin* in lung tissues (n=6). **(F)** Images and **(G)** quantification of immunohistochemistry staining with Col I in lung sections (n=6). Scale bar, 50 μm. **(H)** Images and **(I)** quantification of immunofluorescent staining with Col I in lung sections (n=6). Scale bar, 50 μm. **(J)** Relative mRNA expression of *Col I* in lung tissues (n=6). **(K)** Images and **(L)** quantification of immunohistochemistry staining with collagen III in lung sections (n=6). Scale bar, 50 μm. **(M)** Images and **(N)** quantification of immunofluorescent staining with Col III in lung sections (n=6). Scale bar, 50 μm. **(O)** Relative mRNA expression of *Col III* in lung tissues (n=6). Lung tissues and sections were obtained from *Piezo1^fl/fl^* and *Piezo1^△CDH5^* mice subjected to BLM (14/21 days) or sham operation. Data are presented as mean ± SEM. *P<0.05, **P<0.01, ***P < 0.001, ns, not significant.


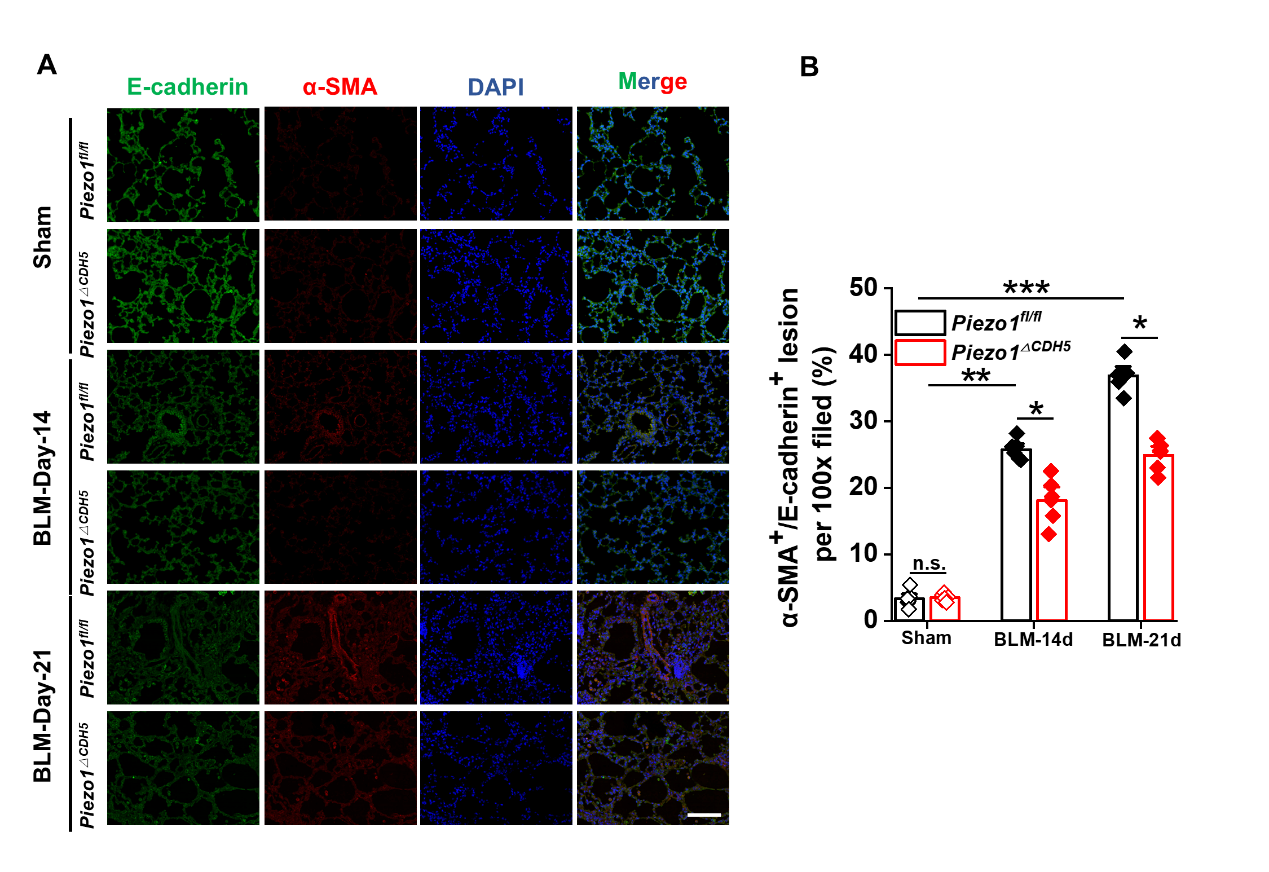


**Figure S4. EC-specific Piezo1 knockout decreases pulmonary fibrosis by inhibiting EMT. (A)** Dual immunofluorescent staining targeting on E-cadherin (green) and α-SMA (red), and **(B)** the quantification in lung sections from *Piezo1^fl/fl^* and *Piezo1^△CDH5^* mice subjected to BLM (14/21 days) or sham operation (n=6). Scale bar, 50 μm. Data are presented as mean ± SEM. *P<0.05, **P<0.01, ***P < 0.001, ****P < 0.0001, ns, not significant.


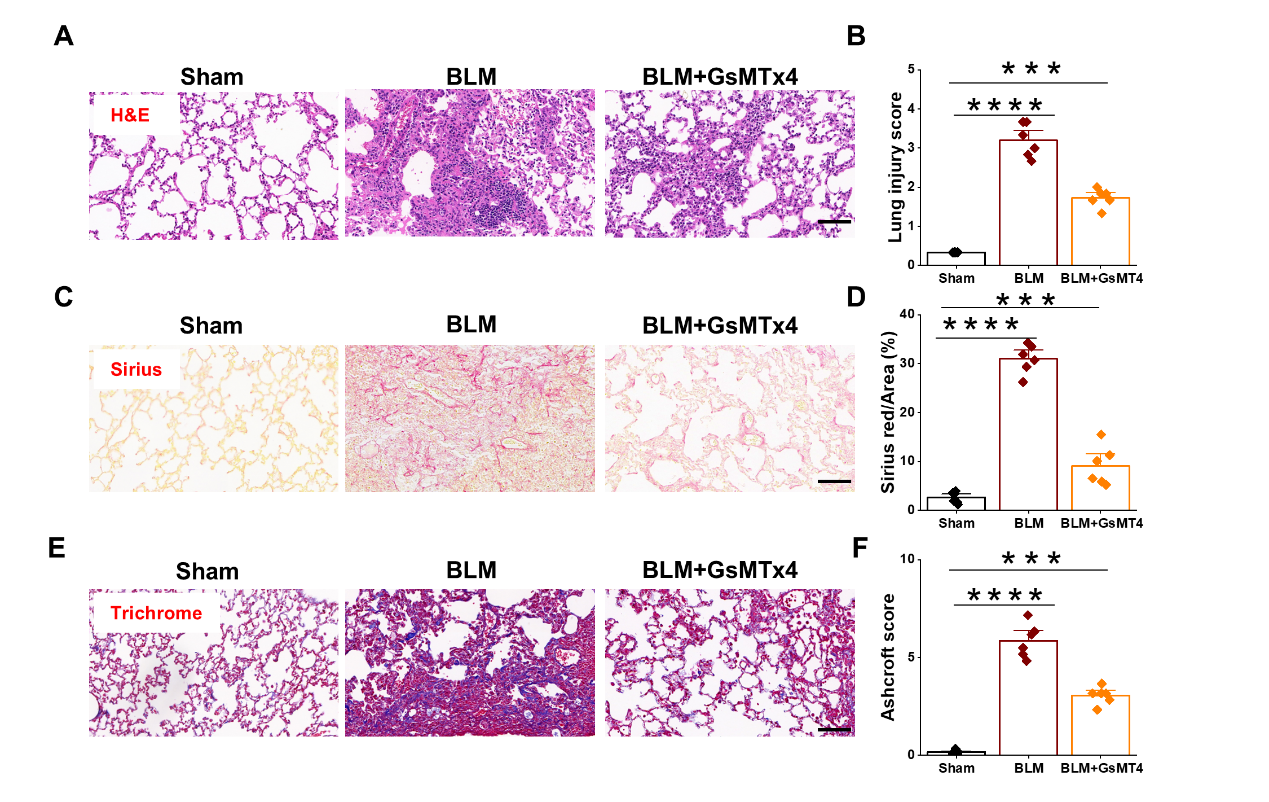


**Figure S5. Inhibition of Piezo1 exerts protective effects on pulmonary fibrosis. (A)** H&E staining images and **(B)** lung injury score of each group (n=6). Scale bar, 50 μm. **(C)** Images and **(D)** quantification of collagen positive areas with Sirius red staining (n=6). Scale bar, 50 μm. **(E)** Masson’s trichrome staining images and **(F)** Ashcroft score (n=6). Scale bar, 50 μm. Data are presented as mean ± SEM. *P<0.05, **P<0.01, ***P < 0.001, ****P < 0.0001, ns, not significant.


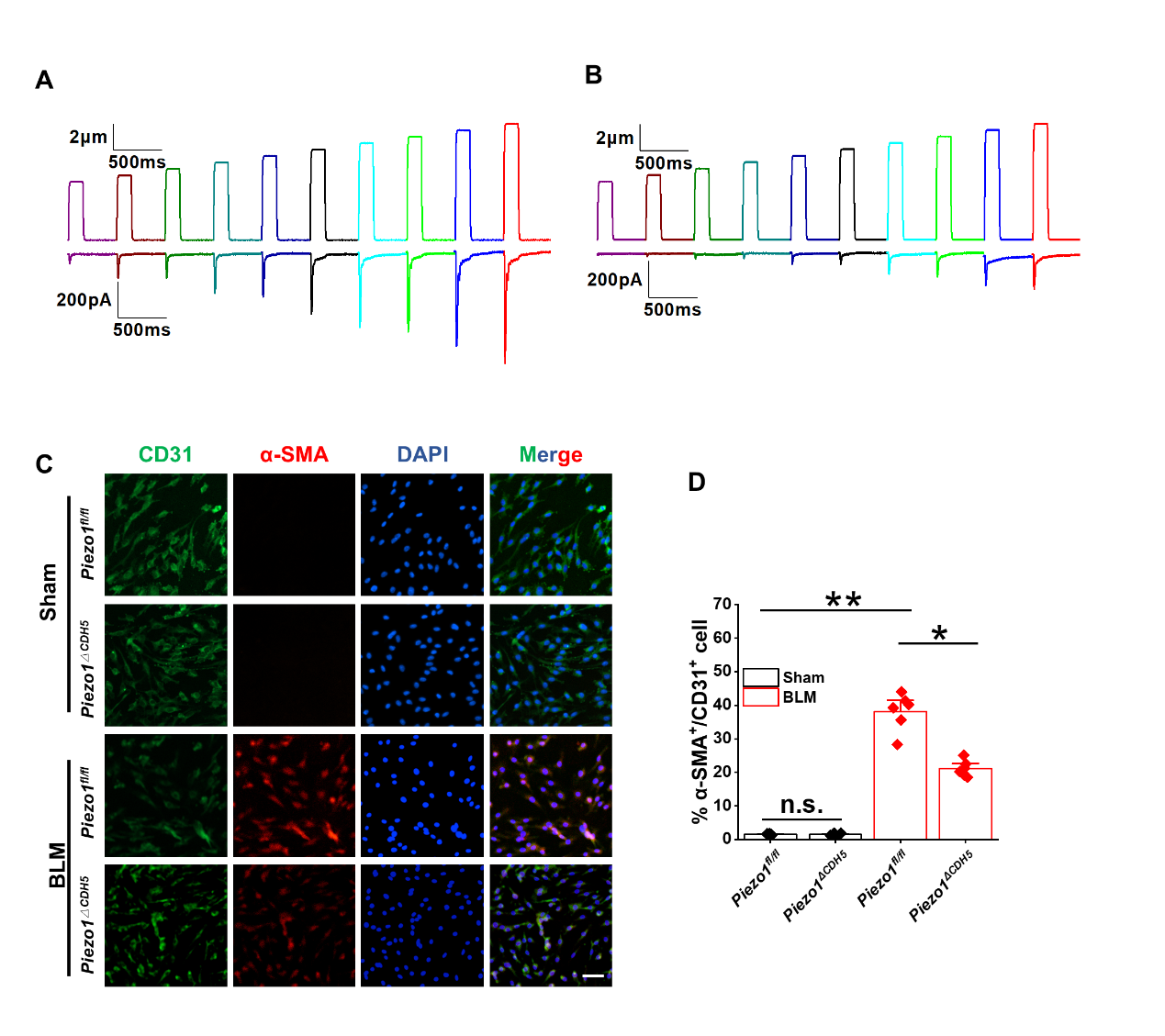


**Figure S6. EMT reduced in PMECs isolated from Piezo1-ECKO mice.** **(A)** Representative traces of current–pressure relationships (I-μm) for currents evoked by mechanical stimulation on PMECs from *Piezo1^fl/fl^* and **(B)** *Piezo1^△CDH5^* mice (n=6). **(C)** Dual immunofluorescent staining targeting on CD31 (green) and α-SMA (red), and **(D)** the quantification in PMECs (n=6). Scale bar, 50 μm. Data are presented as mean ± SEM. *P<0.05, **P<0.01, ns, not significant.


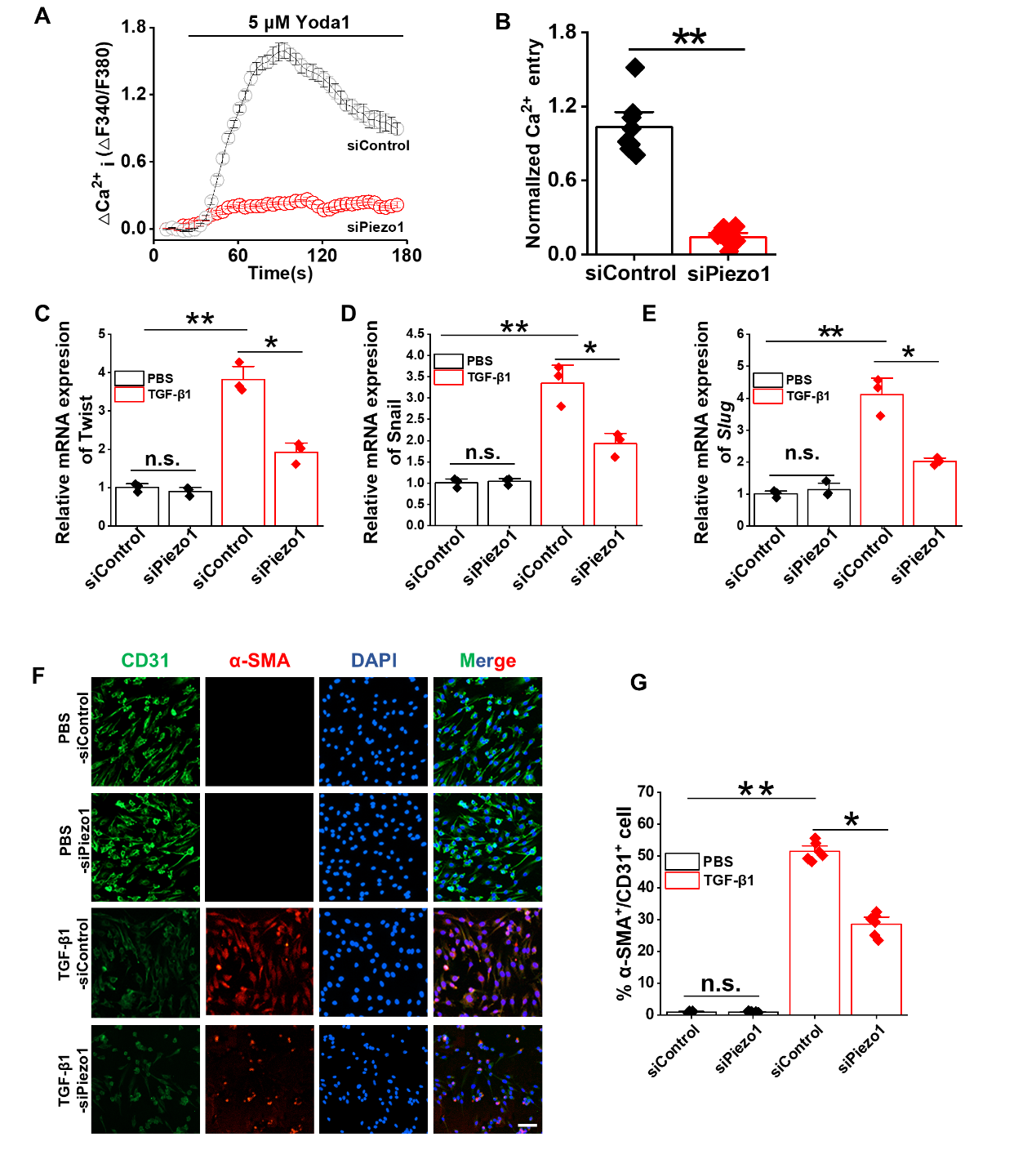


**Figure S7. Piezo1 knockdown alleviates EndMT in vitro. (A)** Measurements of intracellular Ca^2+^ flux and **(B)** peak value in HUVECs with siControl or si*Piezo1* in response to 5 μM Yoda1 (n=8). **(C-E)** Relative mRNA expression of *Twist*, *Snail1* and *Slug* in HUVECs with siControl or si*Piezo1* treated with PBS or TGF-β1 (n=6). **(F)** Dual immunofluorescent staining targeting on CD31 (green) and α-SMA (red), and **(G)** the quantification in HUVECs with siControl or si*Piezo1* treated with PBS or TGF-β1 (n=6). Scale bar, 50 μm. Data are presented as mean ± SEM. *P<0.05, **P<0.01, ns, not significant.


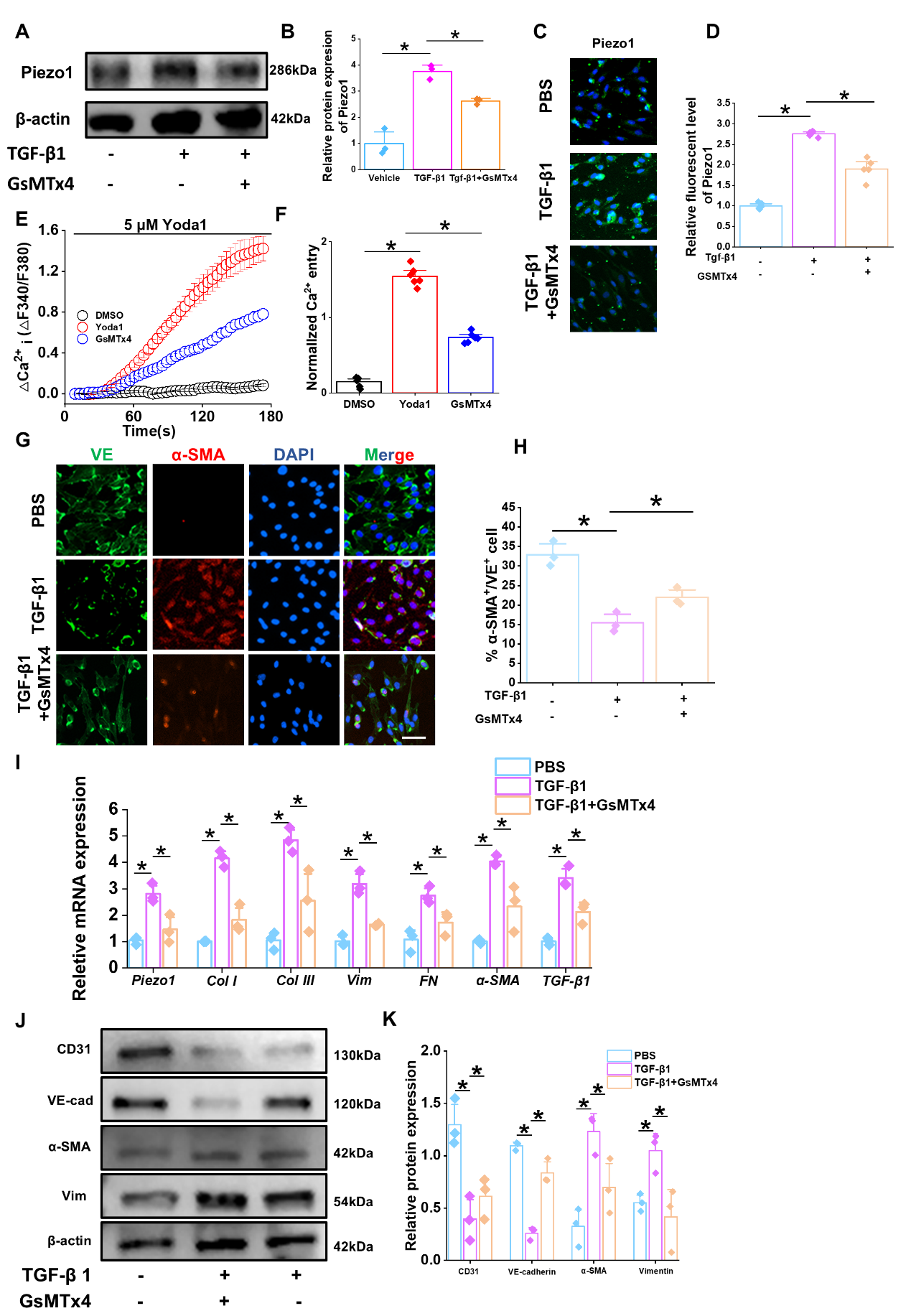


**Figure S8. Piezo1 inhibitor GsMTx4 decreases EndMT induced by TGF-β1 in vitro.** HUVECs were treated by PBS or TGF-β1 or TGF-β1+ GsMTx4. **(A)** Western blot analysis and **(B)** quantification of the protein expression of Piezo1 in HUVECs of each group (n=3). **(C)** Images and **(D)** quantification of immunofluorescent staining with Piezo1 in HUVECs of each group (n=6). **(E-F)** GsMTx4 inhibited the intracellular Ca^2+^ flux induced by Piezo1 activation in HUVECs in response to 5 μM Yoda1 (n=6). **(G)** Images and **(H)** quantification of dual immunofluorescence staining with VE (green) and α-SMA (red) in HUVECs of each group (n=3). Scale bar, 50 μm. **(I)** Relative mRNA expression of *Piezo1*, *Col I*, *Col III*, *Vim*, *FN*, *α-SMA* and *TGF-β1* in HUVECs of each group (n=6). **(J)** Western blot analysis and **(K)** quantitative data of CD31, VE-cad, α-SMA and Vim expression in HUVECs treated by PBS or TGF-β1 or TGF-β1+ GsMTx4 (n=3). Data are presented as mean ± SEM.; *P < 0.05.


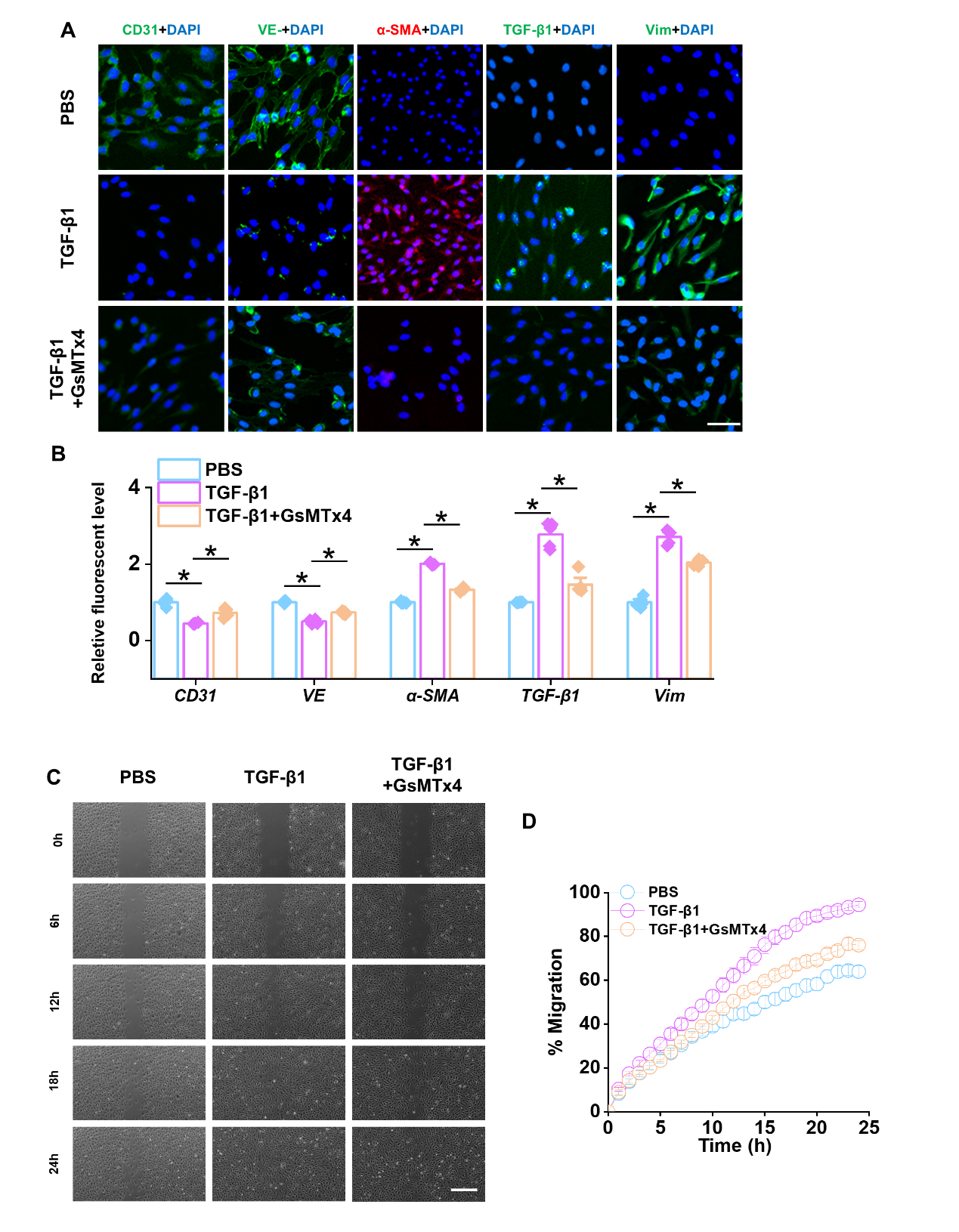


**Figure S9. Endothelial *Piezo1* knockdown inhibited EndMT.** HUVECs were treated by PBS or TGF-β1 or TGF-β1+GsMTx4. **(A)** Images and **(B)** quantitative of immunofluorescence staining with CD31, VE, α-SMA, TGF-β1 and Vim in HUVECs of each group (n=3). Scale bar, 50 μm. **(C)** Images of migration assay and **(D)** percentage of migration in 24h were measured in HUVECs of each group (n=3). Data are presented as mean ± SEM. *P<0.05.


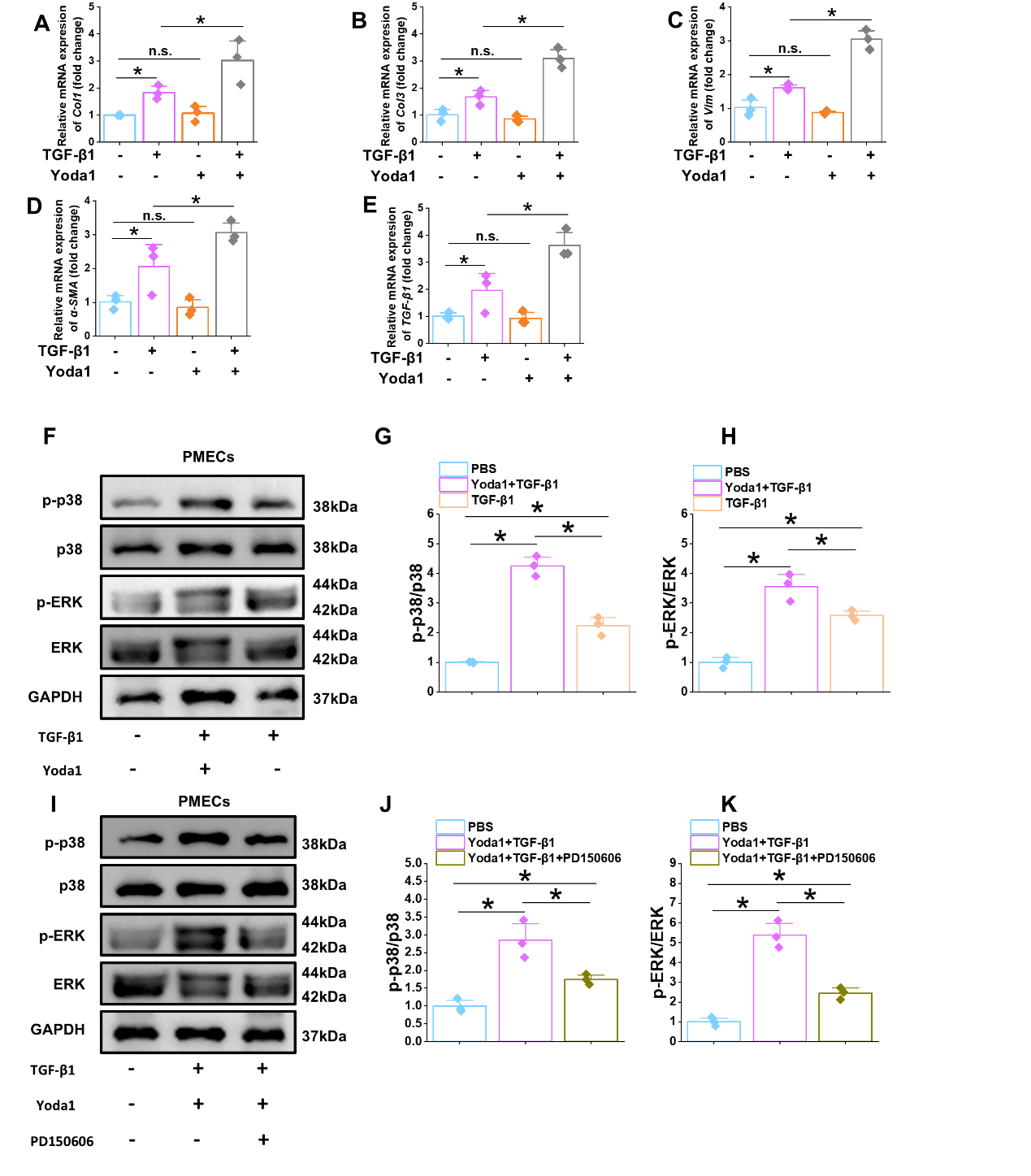


**Figure S10. Piezo1 inhibition decreases phosphorylation of p38-ERK/MAPK through Ca^2+^/Calpain. (A-E)** Relative mRNA expression of *Col I*, *Col III*, *Vim*, *α-SMA* and *TGF-β* in HUVECs treated with or without TGF-β1 and Yoda1 (n=3). **(F)** Western blot analysis and **(G-H)** quantification of the protein expression of p-p38, p38, p-ERK and ERK in PMECs treated with TGF-β1 with or without Yoda1 (n=3). **(I)** Western blot analysis and **(J-K)** quantification of the protein expression of p-p38, p38, p-ERK and ERK in PMECs with TGF-β1+Yoda1 with or without PD150606 (n=3). Data are presented as mean ± SEM. *P<0.05.
