# Supplementary figures and images for: Piezo1 specific deletion in endothelial cell protects the progression of pulmonary fibrosis in mice
Source: Cell Commun Signal. 2026 Feb 28;24:207. doi: 10.1186/s12964-026-02758-7 (PMC13049882; doi:10.1186/s12964-026-02758-7)

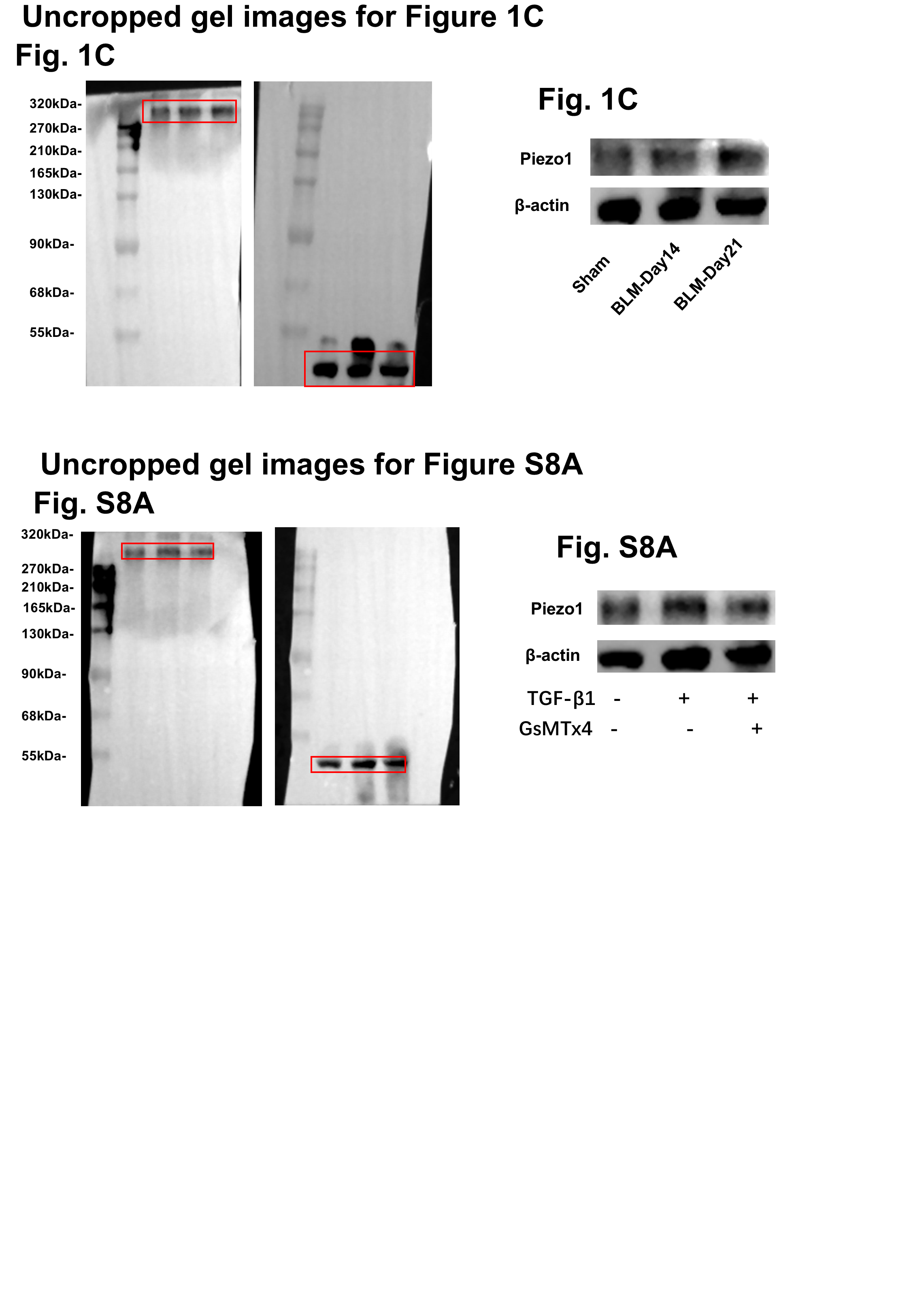

Supplement: Supplementary file 2 — Supplementary Material 2. [file 12964_2026_2758_MOESM2_ESM.zip › WB1.TIF]

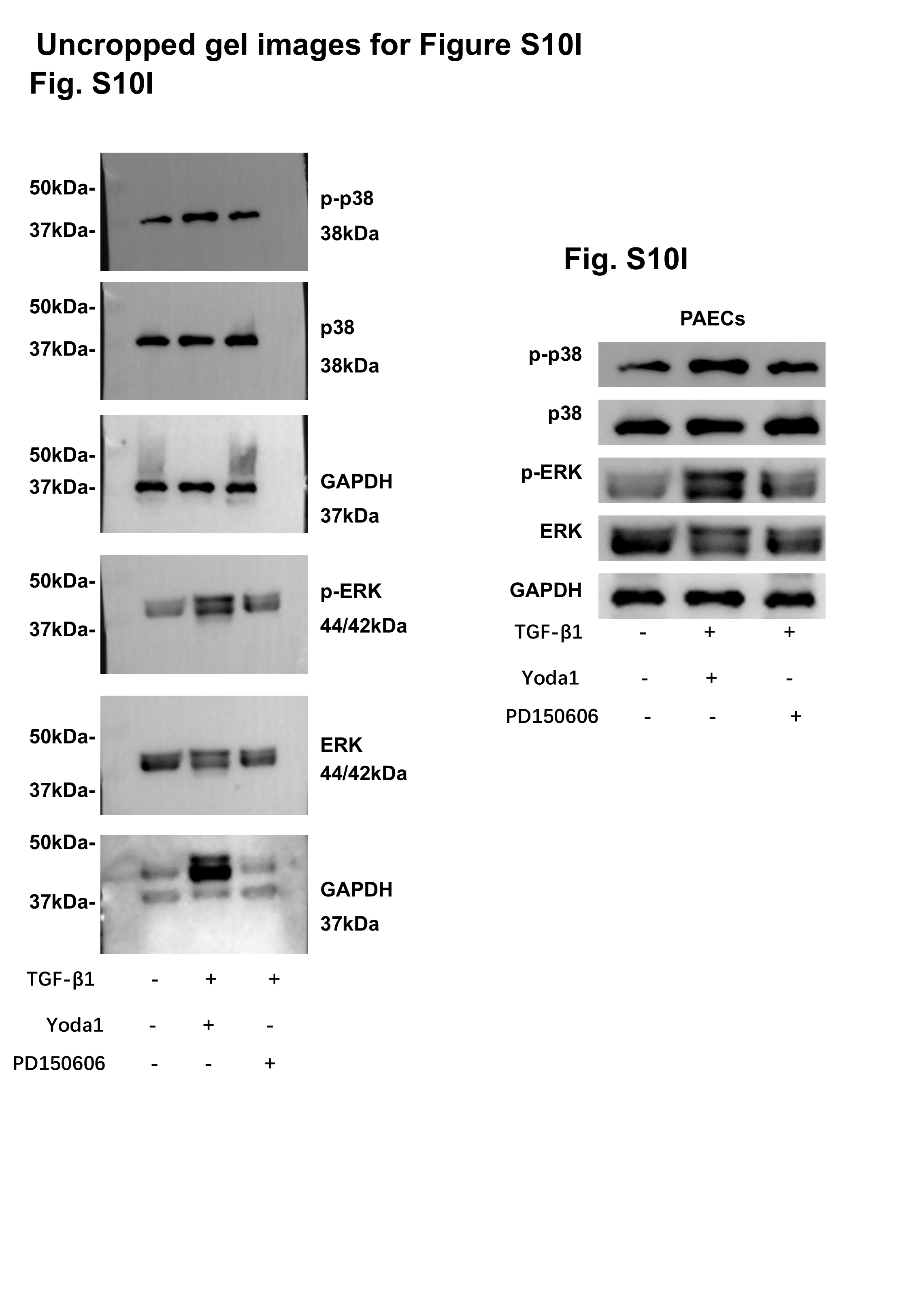

Supplement: Supplementary file 2 — Supplementary Material 2. [file 12964_2026_2758_MOESM2_ESM.zip › WB10.TIF]

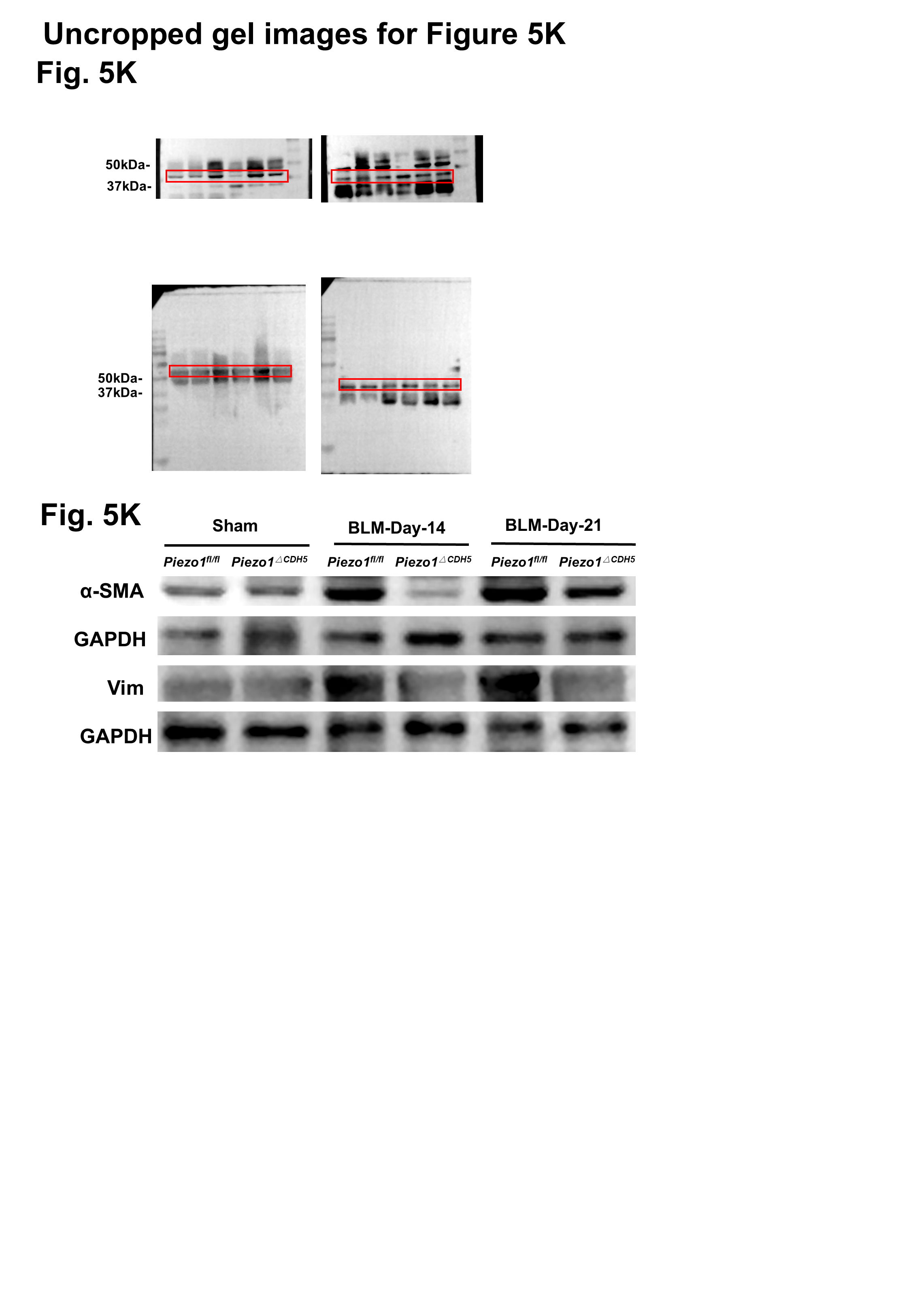

Supplement: Supplementary file 2 — Supplementary Material 2. [file 12964_2026_2758_MOESM2_ESM.zip › WB2.TIF]

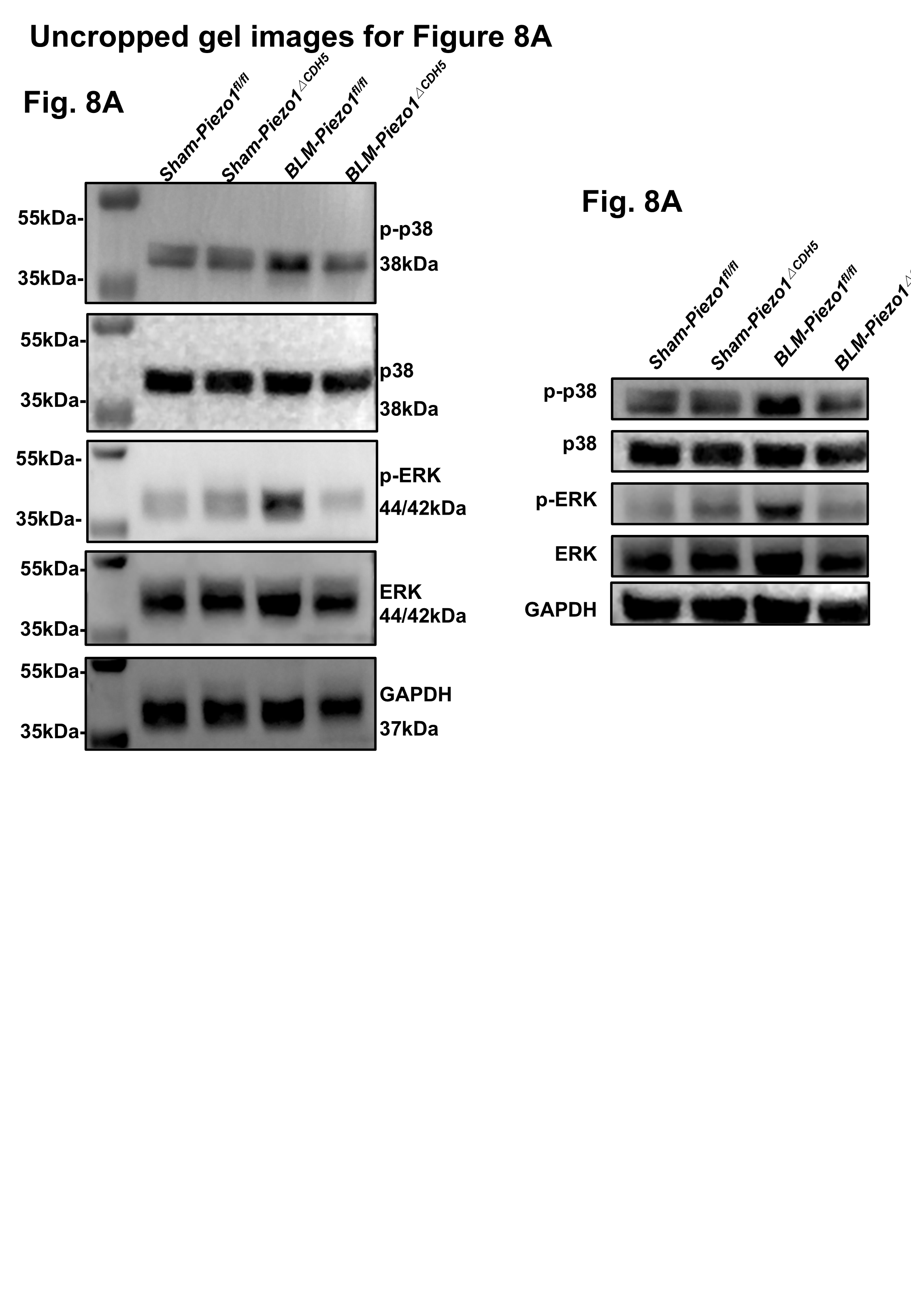

Supplement: Supplementary file 2 — Supplementary Material 2. [file 12964_2026_2758_MOESM2_ESM.zip › WB3.TIF]

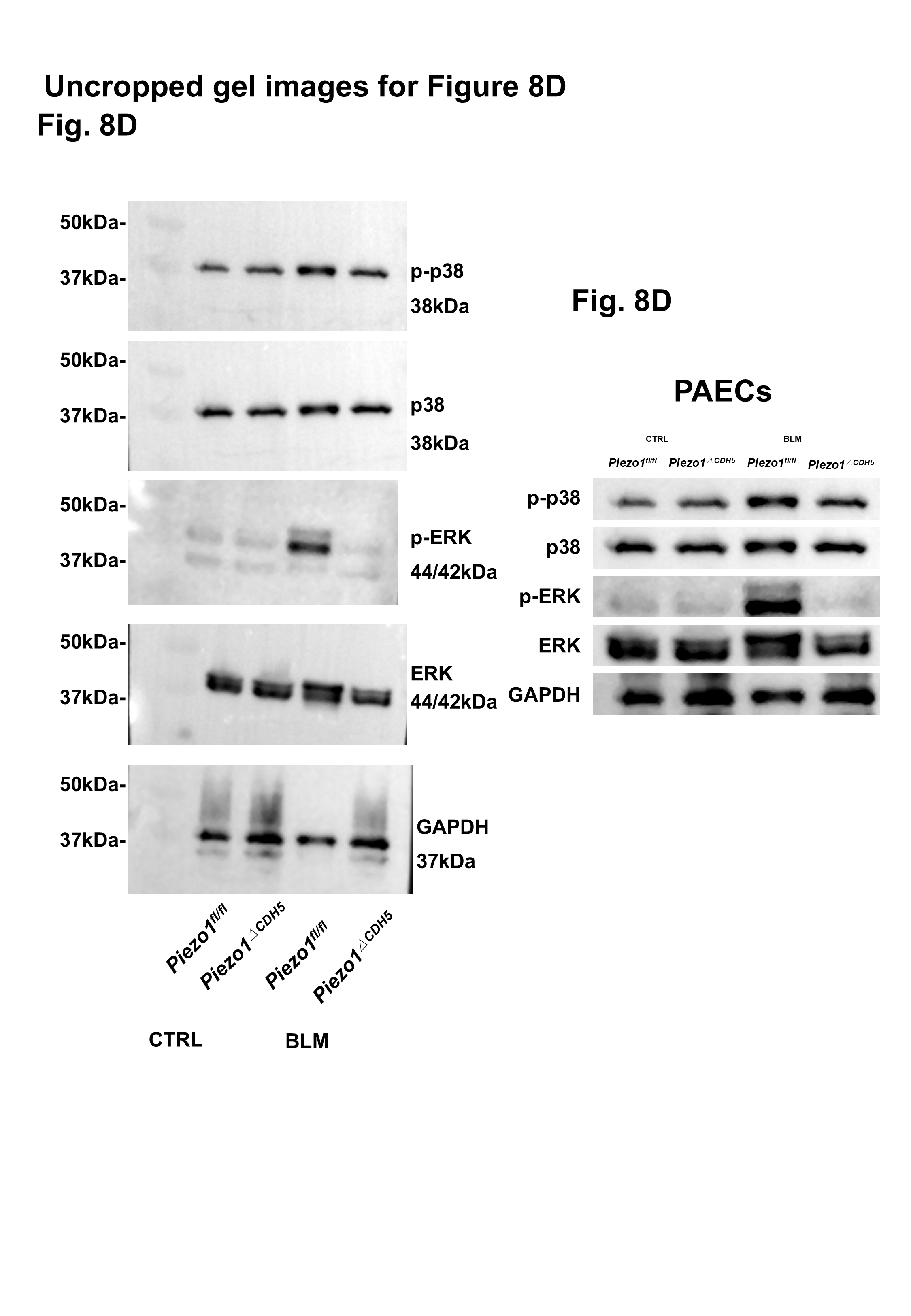

Supplement: Supplementary file 2 — Supplementary Material 2. [file 12964_2026_2758_MOESM2_ESM.zip › WB4.TIF]

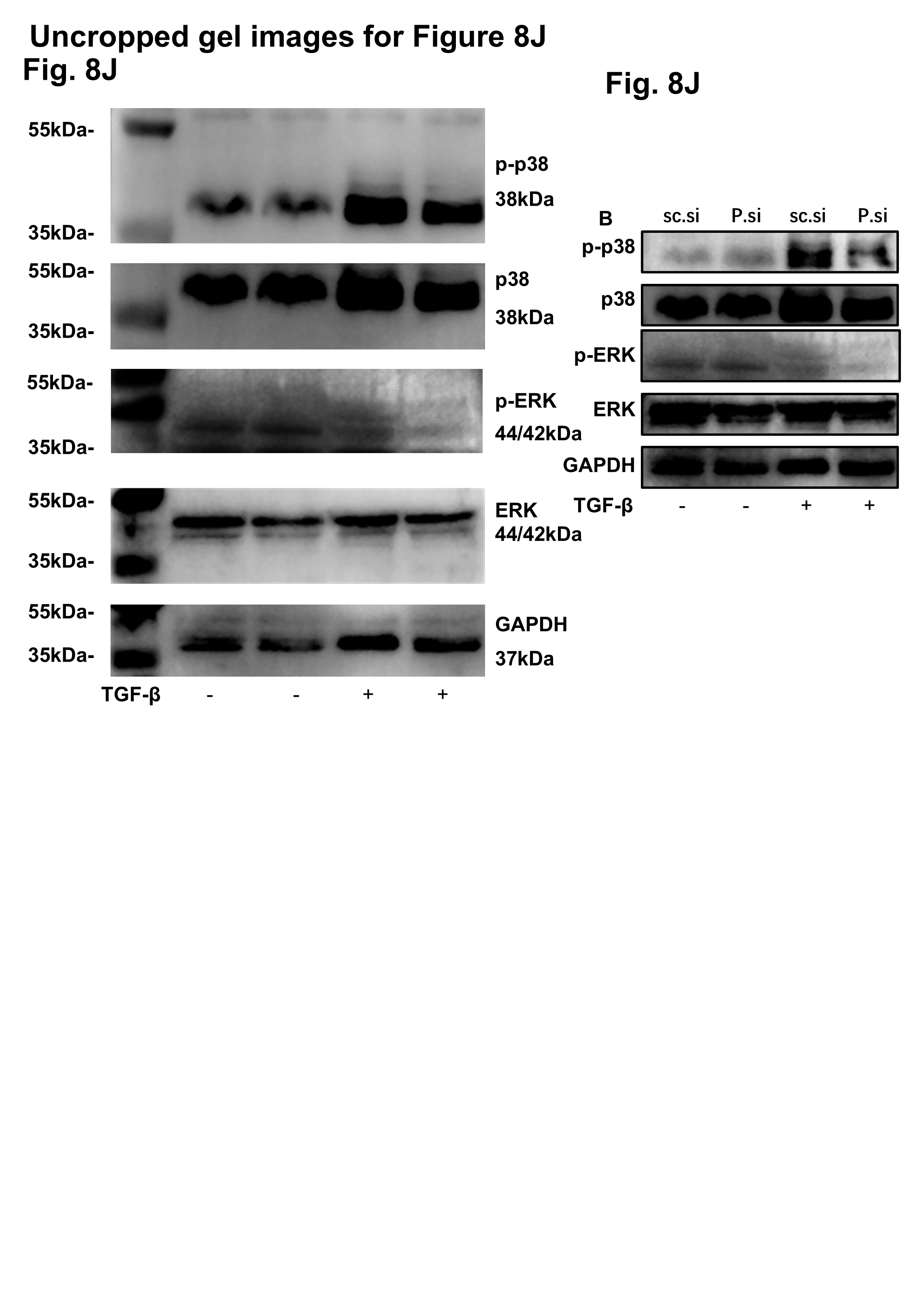

Supplement: Supplementary file 2 — Supplementary Material 2. [file 12964_2026_2758_MOESM2_ESM.zip › WB5.TIF]

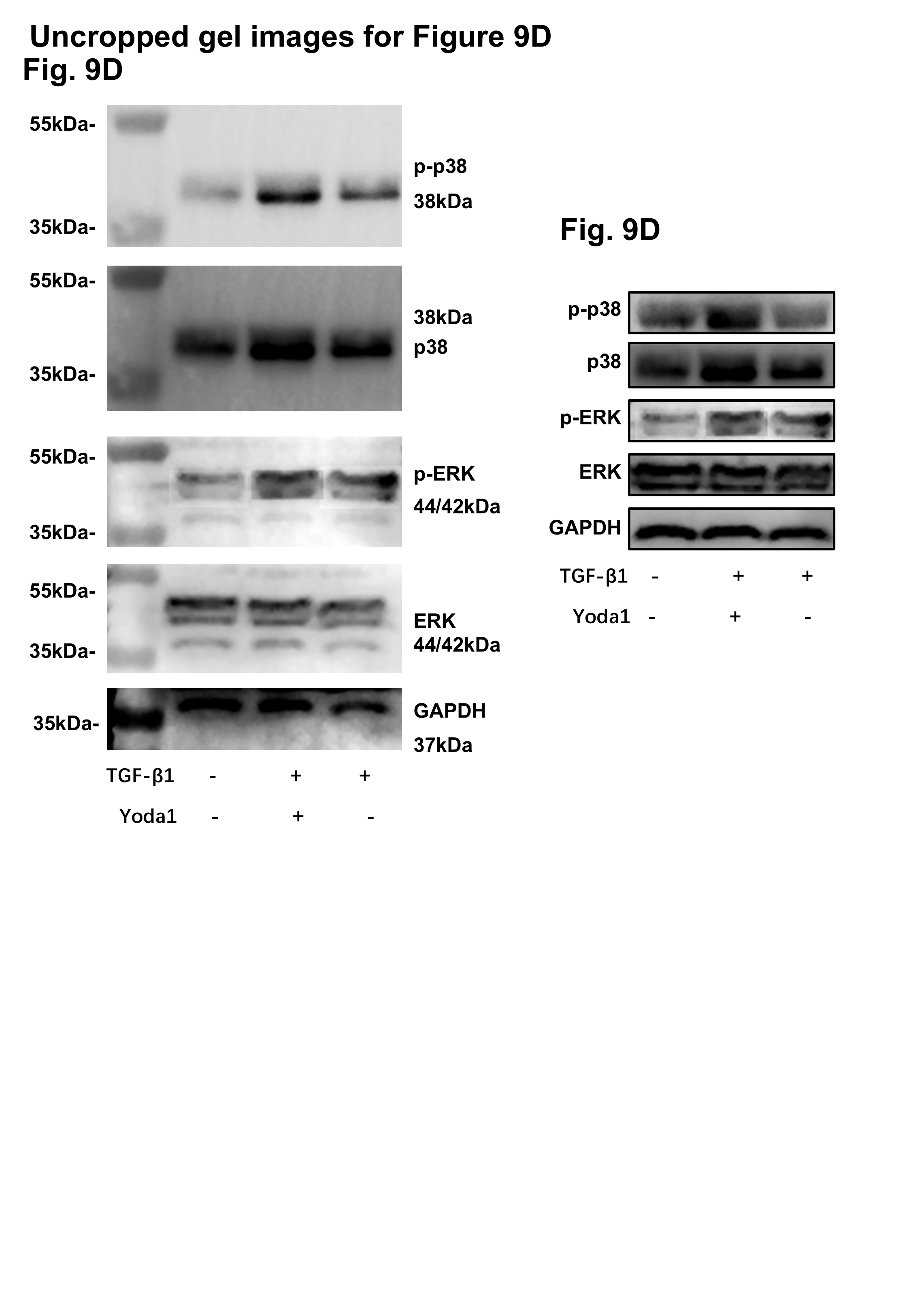

Supplement: Supplementary file 2 — Supplementary Material 2. [file 12964_2026_2758_MOESM2_ESM.zip › WB6.TIF]

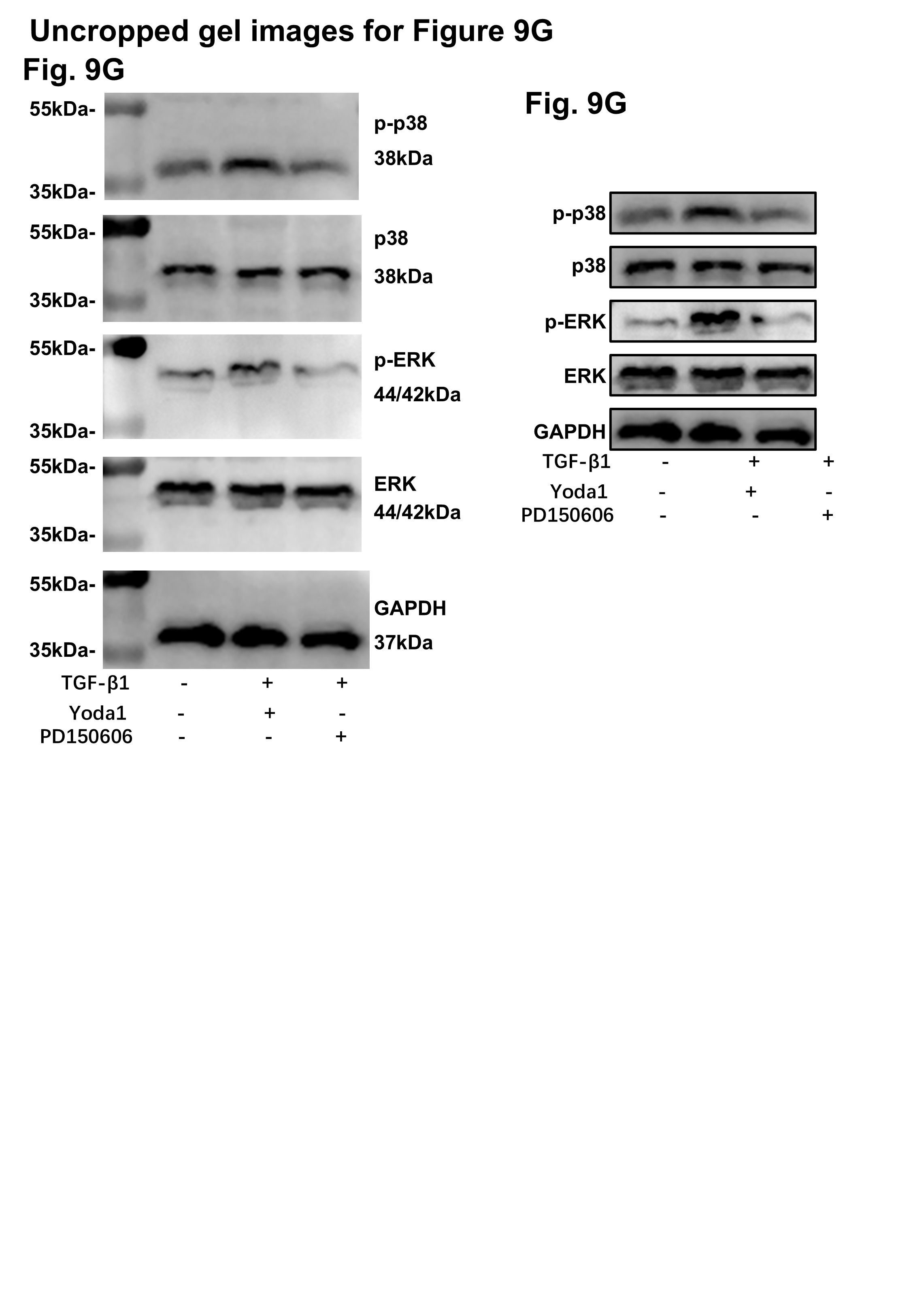

Supplement: Supplementary file 2 — Supplementary Material 2. [file 12964_2026_2758_MOESM2_ESM.zip › WB7.TIF]

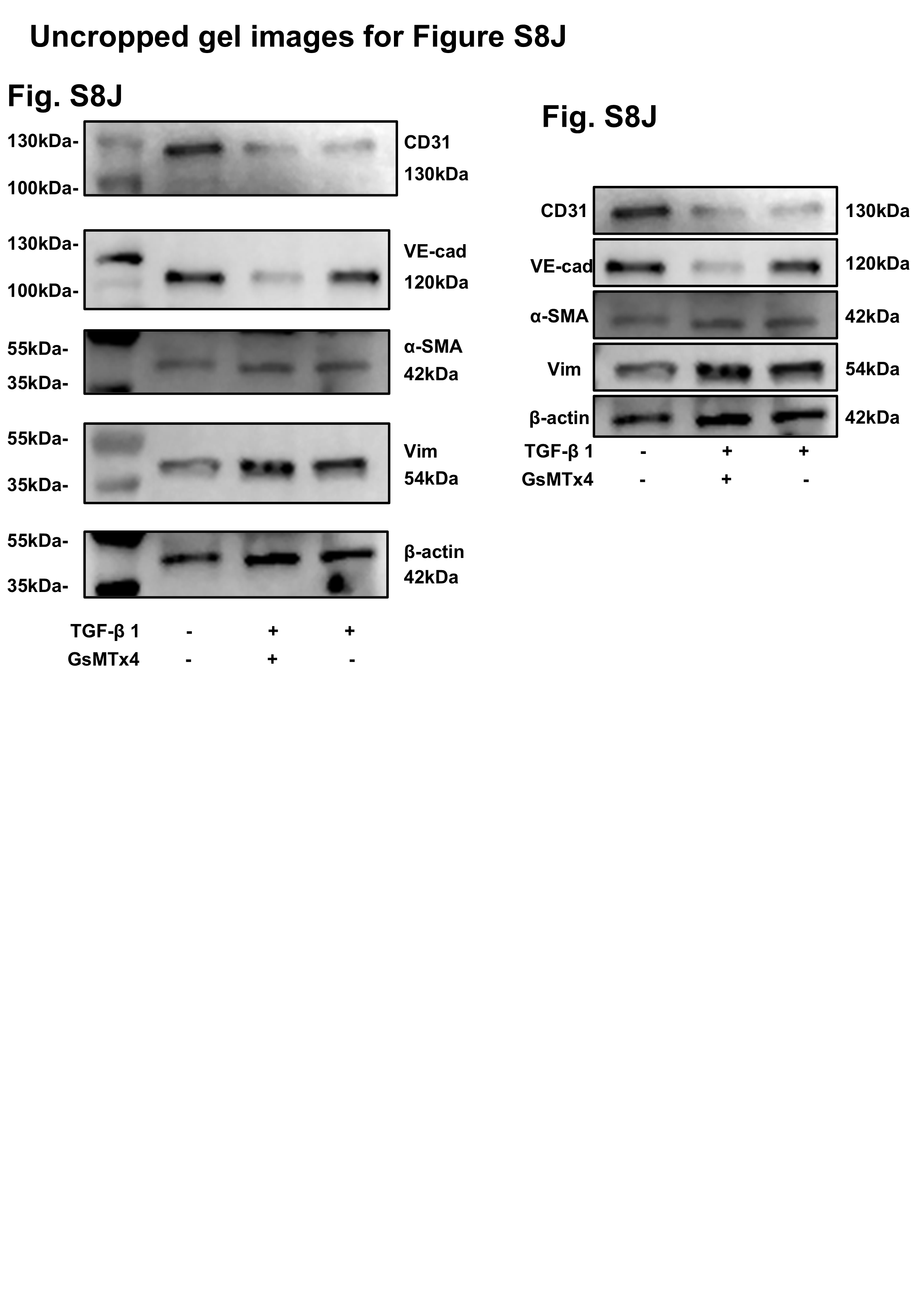

Supplement: Supplementary file 2 — Supplementary Material 2. [file 12964_2026_2758_MOESM2_ESM.zip › WB8.TIF]

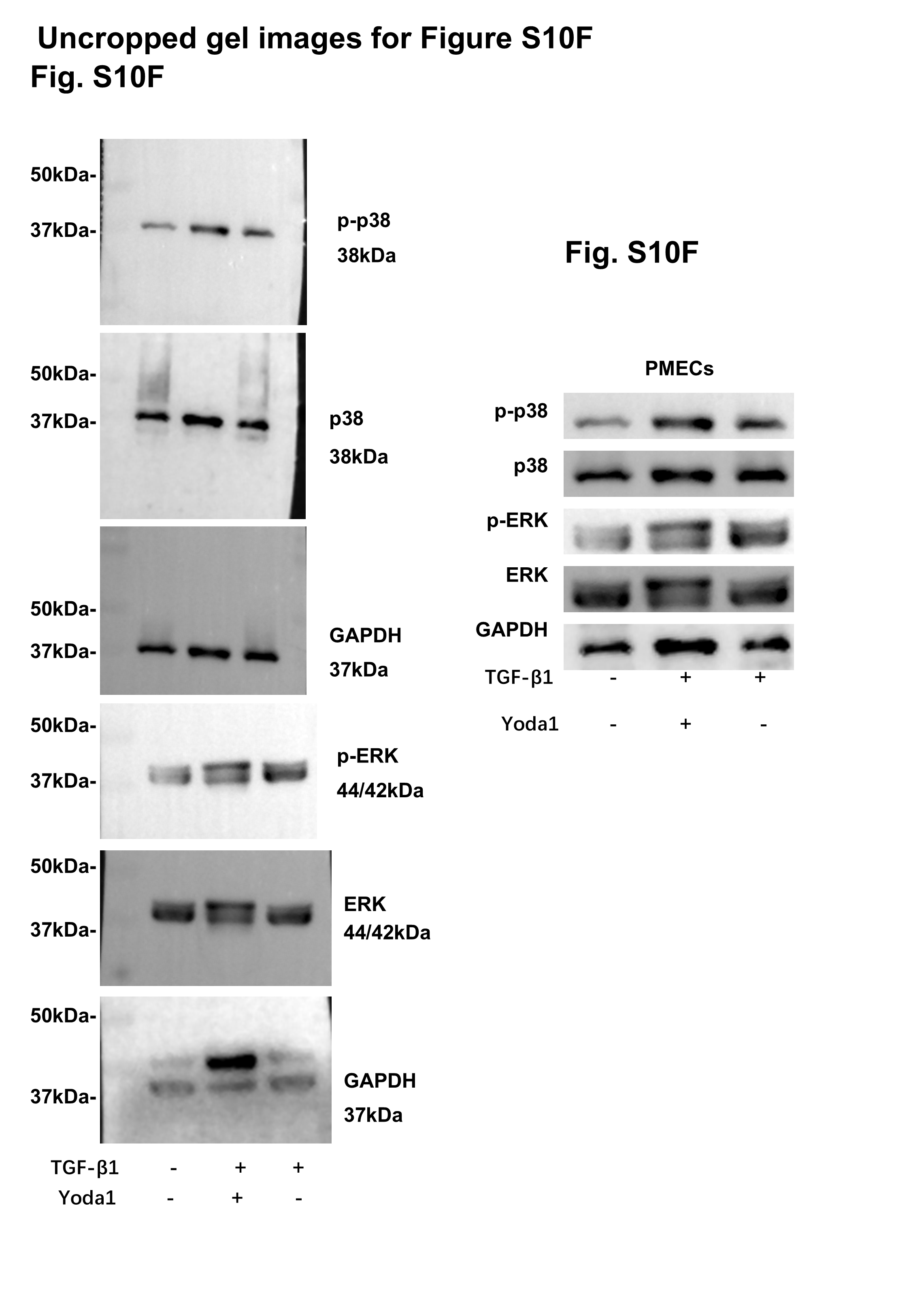

Supplement: Supplementary file 2 — Supplementary Material 2. [file 12964_2026_2758_MOESM2_ESM.zip › WB9.TIF]
